# Boosting the influenza vaccine schedule in children with cancer: a prospective open-label study

**DOI:** 10.1038/s41541-025-01256-0

**Published:** 2025-08-26

**Authors:** Sung K. Chiu, Eliska Furlong, Elizabeth J. McKinnon, Annette Fox, Stephany Sánchez Ovando, Louise Carolan, Andrew McLean-Tooke, Joyce Oommen, Daniel K. Yeoh, Laurence C. Cheung, Nicholas G. Gottardo, Rishi S. Kotecha

**Affiliations:** 1 Leukaemia Translational Research Laboratory, WA Kids Cancer Centre, The Kids Research Institute Australia, Perth, WA Australia; 2 Curtin Medical School, Curtin University, Perth, WA Australia; 3 Department of Clinical Haematology, Oncology, Blood and Marrow Transplantation, Perth Children’s Hospital, Perth, WA Australia; 4 WHO Collaborating Centre for Reference and Research on Influenza, Royal Melbourne Hospital, Peter Doherty Institute for Infection and Immunity, Melbourne, VIC Australia; 5 Department of Infectious Diseases, University of Melbourne, Peter Doherty Institute for Infection and Immunity, Melbourne, VIC Australia; 6 Department of Clinical Immunology, Sir Charles Gairdner Hospital, Perth, WA Australia; 7 Wesfarmers Centre of Vaccines and Infectious Diseases, The Kids Research Institute Australia, Perth, WA Australia; 8 Medical School, University of Western Australia, Perth, WA Australia; 9 Curtin Medical Research Institute, Curtin University, Perth, WA Australia

**Keywords:** Paediatric cancer, Inactivated vaccines

## Abstract

Current immunization guidelines recommend one dose of influenza vaccine for children aged ≥9 years and two doses for younger or vaccine-naïve children. However, children receiving chemotherapy have an attenuated immune response. We performed a prospective open-label study in children undergoing treatment for cancer at Perth Children’s Hospital, Western Australia, to examine the safety and efficacy of a boosted influenza schedule. This comprised three vaccine doses for children <9 years of age and two doses for those ≥9 years, with each dose administered at least 4 weeks apart. The additional vaccine dose was well-tolerated with no serious adverse events reported; it also resulted in improved geometric mean antibody titres for A/H1N1 (70 to 97, *p* = 0.003), A/H3N2 (76 to 104, *p* = 0.003) and B/Washington (148 to 179, *p* = 0.03) strains. In summary, a boosted influenza vaccine schedule is safe and improves humoral immune response, providing a readily implementable strategy to protect children undergoing treatment for cancer.

## Introduction

Influenza is a common seasonal infection associated with significant morbidity, mortality and prolonged hospitalisation among children undergoing treatment for cancer due to the underlying disease and treatment induced immunosuppression^1–4^. These patients are not only more susceptible to the influenza virus compared to healthy children, but often suffer from a prolonged and more severe course of illness, with associated delays in chemotherapy^3–5^. Complications such as bacterial infection have been reported in up to 15% of children with cancer suffering from an influenza infection, further impacting the course of recovery^4^.

Currently, all children in Australia aged 6 months to 18 years undergoing immunosuppressive therapy, including treatment for cancer, are recommended to receive inactivated influenza vaccine as per recommendations developed by the Australian Technical Advisory Group on Immunisation (ATAGI), approved by the National Health and Medical Research Council (NHMRC) and supported by a number of international studies^1^. However, an attenuated immune response to influenza vaccines in children receiving treatment for cancer has been identified, necessitating strategies to improve immunogenicity^1^. A number of methods have been evaluated, including use of high-dose or adjuvanted vaccines to augment antibody response, to varying effect^1,6–8^. Previously, we identified that two doses of the trivalent inactivated influenza vaccine in children under 10 years of age resulted in an improved antibody response compared to those that received one dose^9^.

To further assess the benefit of repeated vaccination, we sought to determine the safety, feasibility and immunogenicity of adding an extra influenza vaccine dose to the standard recommended schedule in children receiving treatment for cancer.

## Results

### Patient characteristics

There were 62 patients (Supplementary Fig. 1 and Table 1), with 40% recruited in the 2020 winter influenza season and 60% in 2021. The majority of patients were male (71%; *n* = 44/62), diagnosed with a haematological malignancy (65%; *n* = 40/62) and were receiving intensive treatment (74%; *n* = 46/62) as defined in Supplementary Table 1. A history of previous influenza vaccination was reported in 61% participants (*n* = 38/62), with an equal distribution between those <9 (*n* = 20/33) and ≥9 (*n* = 18/29) years of age. At study enrollment, 81% (*n* = 50/62) had lymphopenia and 37% (*n* = 23/62) had low baseline IgG levels.

**Table 1:** Characteristics of patients enrolled onto the study

		Overall *n* = 62	<9 years *n* = 33	≥9 years *n* = 29
Year	2020	25 (40%)	14 (42%)	11 (38%)
	2021	37 (60%)	19 (58%)	18 (62%)
Median age (IQR)		8.5 (4.9–13.7)	5.0 (3.3–6.8)	13.7 (11.2–15.2)
Sex^a^	Male	44 (71%)	24 (73%)	20 (69%)
	Female	18 (29%)	9 (27%)	9 (31%)
Tumour type				
Haematological		40 (65%)	25 (76%)	15 (52%)
Solid organ		22 (35%)	8 (24%)	14 (48%)
Treatment intensity				
High		46 (74%)	24 (73%)	22 (76%)
Low		16 (26%)	9 (27%)	7 (24%)
Previous vaccine				
Yes		38 (61%)	20 (61%)	18 (62%)
No		24 (39%)	13 (39%)	11 (38%)
Lymphocyte count				
Normal		12 (19%)	9 (27%)	3 (10%)
Low		50 (81%)	24 (73%)	26 (90%)
CD19 B cell count				
Normal		13 (21%)	4 (12%)	9 (31%)
Low		49 (79%)	29 (88%)	20 (69%)
CD3 T cell count				
Normal		15 (24%)	6 (18%)	9 (31%)
Low		47 (76%)	27 (82%)	20 (69%)
CD4 T cell count				
Normal		13 (21%)	5 (15%)	8 (28%)
Low		49 (79%)	28 (85%)	21 (72%)
CD8 T cell count				
Normal		20 (32%)	10 (30%)	10 (34%)
Low		42 (68%)	23 (70%)	19 (66%)
Natural killer cell count				
Normal		26 (42%)	11 (33%)	15 (52%)
Low		36 (58%)	22 (67%)	14 (48%)
Immunoglobulin G level				
Normal		39 (63%)	18 (55%)	21 (72%)
Low		23 (37%)	15 (45%)	8 (28%)

*IQR* interquartile range.

^a^denotes sex assigned at birth.

### Safety of the boosted vaccine schedule

**Fig. 1:**
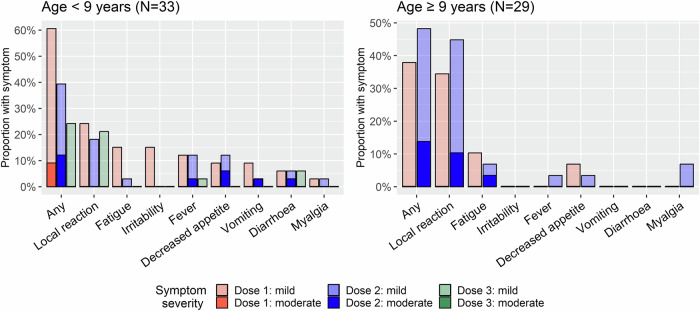
Adverse events following each vaccination dose.

The vaccination schedule was safe and well-tolerated, with all patients completing their vaccination schedule as planned. Adverse events were self-limiting, classified as mild (<9 years of age: 51.5% following dose one, 27.3% following dose two and 24.2% following dose three; ≥9 years of age: 37.9% following dose one and 34.4% following dose two) or moderate (<9 years of age: 9% following dose one, 12.1% following dose two and 0% following dose three; ≥9 years of age: 0% following dose one and 13.8% following dose two), with no severe adverse events recorded (Fig. 1). The most common side effect was local reaction at the site of injection. Children younger than nine years had more systemic side effects, particularly fever, compared to older children. Adverse events following the additional vaccination (second dose for children ≥9 years of age and third dose for children <9 years of age) were similar to those experienced with the standard recommended schedule; notably there were fewer adverse events reported following the third influenza vaccine dose in children <9 years of age compared to those reported following the first two doses (Fig. 1).

### Immunogenicity of the boosted vaccine schedule

**Fig. 2:**
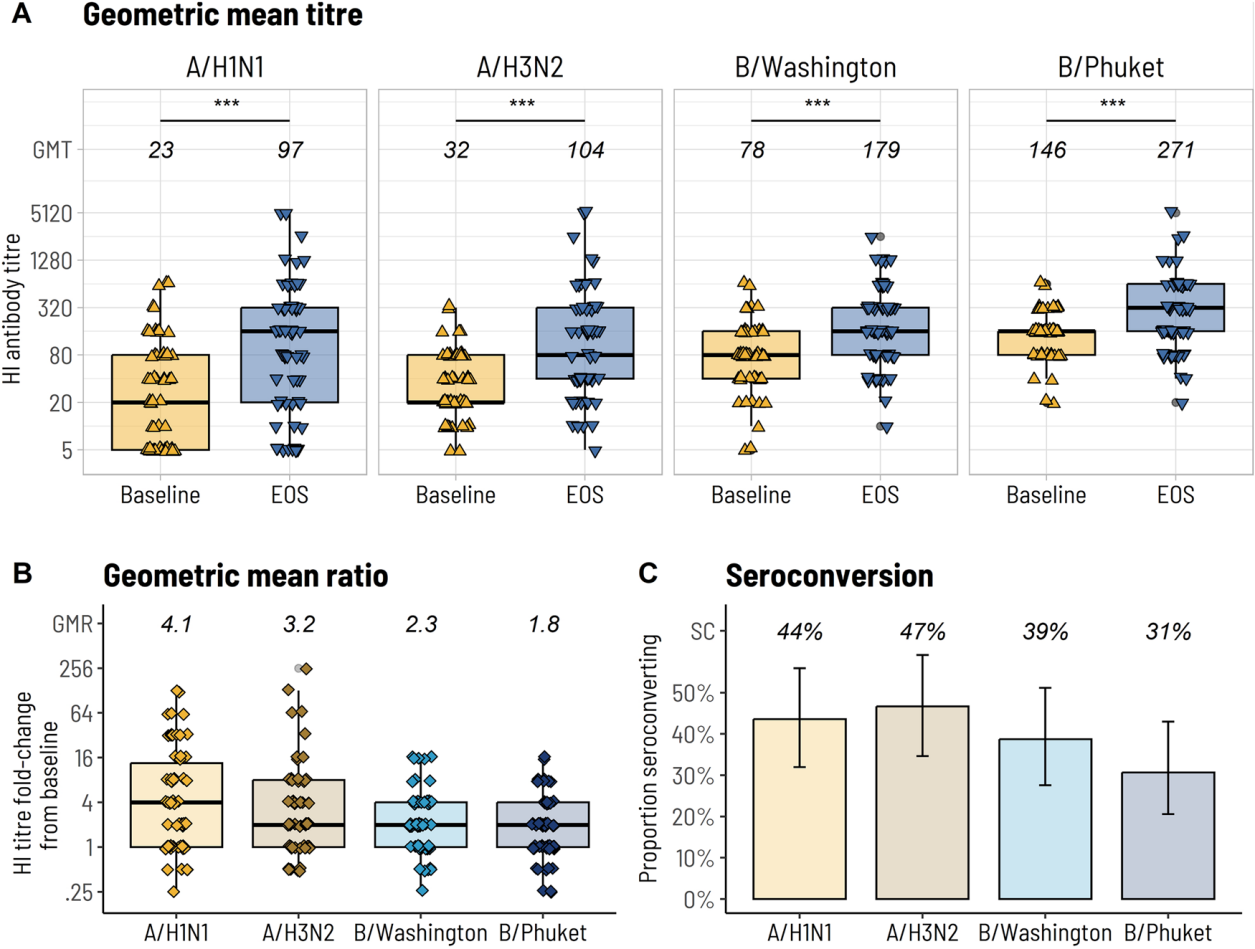
Hemagglutination inhibition antibody response comparison between baseline and end of study. **A** Geometric mean titre, **B** Geometric mean ratio, and **C** Seroconversion rates. ***p < 0.001, paired-sample *t*-test of log-transformed titres. The bolded line of each box plot denotes the median hemagglutination inhibition (HI) antibody titre and the upper and lower bounds denote upper and lower quartiles. Ι bars indicate 95% confidence intervals. EOS end of study.

Hemagglutination inhibition (HI) antibody titres were measured against both egg-grown and cell-grown antigens; we found there was strong correlation between the two methods (Supplementary Fig. 2), with cell-based data reported herein. Prior to vaccination, median geometric mean titre (GMT) was 23.4 (95% Confidence Interval (CI) 15.7–34.8) for A/H1N1, 31.9 (95% CI 25.0–40.6) for A/H3N2, 78.2 (95% CI 61.1–100.1) for B/Washington and 146·3 (95% CI 123.4–173.4) for B/Phuket (Fig. 2). Following completion of the vaccination schedule, median GMT increased significantly from baseline for all four influenza strains in our patient cohort to 97.0 (95% CI 60.0–155.7, *p* < 0.001) for A/H1N1, 104.3 (95% CI 69.3–157.1, *p* < 0.001) for A/H3N2, 178.9 (95% CI 133.3–240.1, *p* < 0.001) for B/Washington and 270.6 (95% CI 206.6–354.4, *p* < 0.001) for B/Phuket. The geometric mean ratio (GMR) relative to baseline was highest for A/H1N1 at 4.14 (95% CI 2.79–6.14), followed by A/H3N2 at 3.21 (95% CI 2.24–4.60), B/Washington at 2.29 (95% CI 1.79–2.91) and lowest for B/Phuket at 1.85 (95% CI 1.44–2.38). Seroconversion rates were 43.5% (95% CI 31.9–55.9) for A/H1N1, 46.7% (95% CI 34.6–59.1) for A/H3N2, 38.7% (95% CI 27.6–51.2) for B/Washington and 30.6% (95% CI 20.6–43.0) for B/Phuket.

**Fig. 3:**
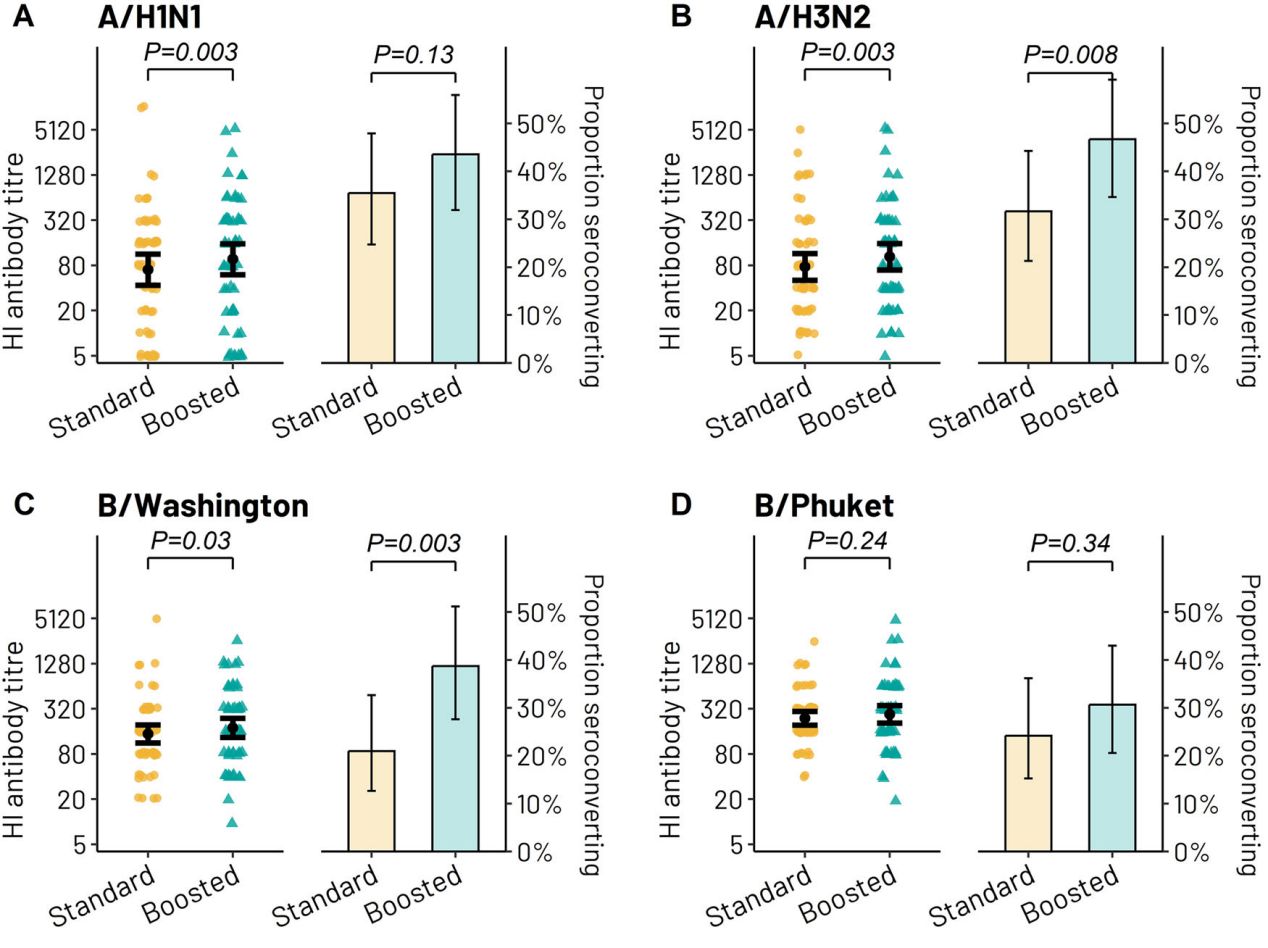
Comparisons of immune response as elicited by the standard (post penultimate dose) and the boosted (post final dose) vaccine schedule. Immune response to **A** A/H1N1, **B** A/H3N2, **C** B/Washington, and **D** B/Phuket. For each panel, time-specific raw data annotated by respective geometric mean titres for hemagglutination inhibition (HI) is shown on the left and time-specific seroconversion rates on the right. *p* values are derived from a paired-sample *t*-test of the log-transformed titres on the left and from a McNemar test of paired proportions on the right. Ι bars indicate 95% confidence intervals.

In order to understand the effect of the additional vaccination dose on antibody response in our patient population, we compared the influenza vaccine response between the boosted and the standard vaccination schedule. There was a significant increase in GMT to both influenza A strains with the additional vaccine dose (A/H1N1: 70 to 97, *p* = 0.003; A/H3N2: 76 to 104, *p* = 0.003), as well as B/Washington (148 to 179, *p* = 0.03), whereas the improvement with the additional vaccine dose to B/Phuket was not statistically significant (239 to 271, *p* = 0.24) (Fig. 3). The corresponding incremental effect on GMR with the additional vaccine dose was 1.38 (95% CI 1.12–1.71) for A/H1N1, 1.37 (95% CI 1.11–1.68) for A/H3N2, 1.21 (95% CI 1.02–1.44) for B/Washington and 1.13 (95% CI 0.92–1.39) for B/Phuket. The percentage of patients that seroconverted following the boosted versus standard vaccine schedule was 43.5% vs. 35.5% for A/H1N1 (*p* = 0.13), 46.7% vs. 31.7% for A/H3N2 (*p* = 0.008), 38.7% vs. 21.0% for B/Washington (*p* = 0.003) and 30.6% vs. 24.2% for B/Phuket (*p* = 0.34) (Fig. 3).

**Fig. 4:**
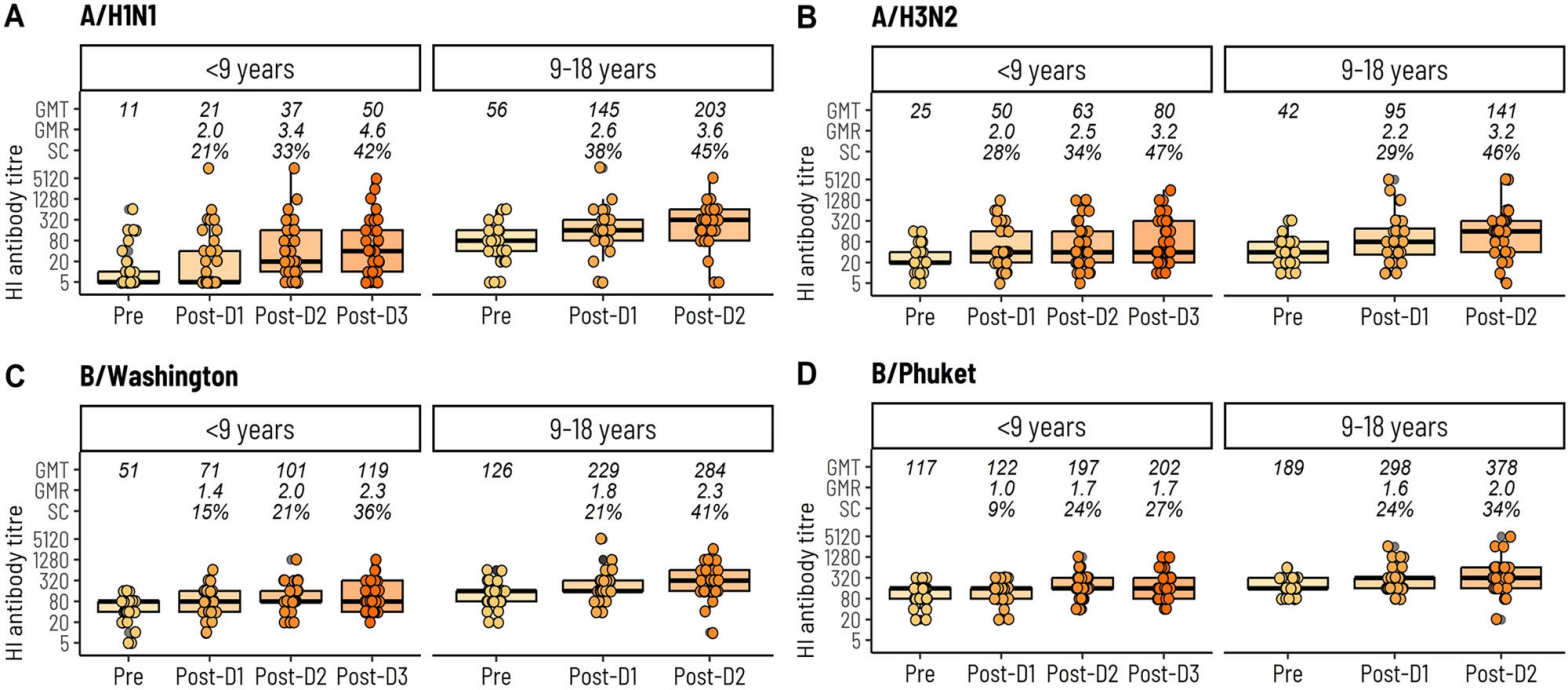
Hemagglutination inhibition antibody titres at baseline and after each influenza vaccine according to age group. Hemagglutination inhibition (HI) antibody titres to **A** A/H1N1, **B** A/H3N2, **C** B/Washington, and **D** B/Phuket. GMT geometric mean titre, GMR geometric mean ratio relative to baseline, SC seroconversion. The bolded line of each box plot denotes the median HI antibody titre and the upper and lower bounds denote upper and lower quartiles.

Age was the only clinical characteristic that was consistently associated with antibody titres (Fig. 4 and Supplementary Table 2). Antibody titres at baseline for all four influenza strains were consistently lower for patients aged <9 years compared to those aged ≥9 years; these age-related differences remained significant upon completion of vaccination for A/H1N1, B/Washington and B/Phuket. Nevertheless, for all four influenza strains, there were no significant differences in the GMR between the two age groups at the end of the study indicating that age did not impact on the overall vaccine response (Supplementary Table 2).

## Discussion

Children with cancer undergoing chemotherapy are at increased risk of serious sequelae from influenza; however, there are few studies that have specifically assessed immunisation to prevent influenza in this population and the optimal vaccine dose or schedule is unknown^1^. Our study is the first to demonstrate that a boosted vaccination schedule, comprising two doses of the quadrivalent inactivated influenza vaccine for children ≥9 years of age and three doses for those <9 years, is safe and can improve antibody response in immunocompromised children receiving treatment for cancer compared to the standard schedule.

At the end of the study, there was a significant increase in antibody titres from baseline to all four influenza strains, with the additional dose contributing to improvements in immunogenicity to the A/H1N1, A/H3N2 and B/Washington strains. The increase in antibody titre with the last dose of influenza vaccine as measured by GMR was 38% for A/H1N1, 37% for A/H3N2 and 21% for B/Washington. Younger age was the only clinical characteristic that was associated with reduced antibody titres both at baseline and at the end of study; nevertheless there were no age-specific differences in GMR following the immunisation schedule suggesting that the additional vaccine dose was beneficial for all children undergoing cancer treatment. Our results are consistent with recent studies demonstrating improved immune response to an additional dose of the COVID-19 vaccine in immunosuppressed patients^10^.

Our findings occurred despite relatively high baseline antibody titres in our patient cohort, especially for influenza B strains, compared to our previous study conducted in 2010–2011^9^. This may be in part due to the high uptake of preceding influenza vaccination in our study participants in the context of a government-funded influenza vaccine programme for all children in Australia^11,12^, as well as less genetic change in influenza B viruses; for example the B/Phuket strain has been targeted in the quadrivalent influenza vaccine in Australia since 2017. This may provide reasoning as to why the serological improvement to B/Phuket with the additional vaccine dose was not found to be statistically significant. For further context, since the COVID-19 pandemic, there has been disappearance of reported cases of influenza caused by B/Yamagata lineage, which includes the sub-lineage B/Phuket, leading to consideration of removal of B/Yamagata lineage from future influenza vaccines^13–15^.

In addition to improving the humoral immune response, we found that boosting the influenza immunisation schedule with an additional dose was safe and well tolerated, with a comparable adverse event profile to recent publications which used the standard schedule in this patient population^8,9,16,17^. The importance of this safety profile cannot be under-estimated given that a common reason for vaccine hesitancy is fear of side effects^18^. Strong recommendations and positive attitudes of health care providers have also been shown to influence vaccine uptake and thus education of oncologists and primary care physicians will be an important consideration for implementation of a boosted schedule in children with cancer^19^.

There are several limitations of this study. Despite an attenuated humoral antibody response, two studies which assessed children post-haematopoietic stem cell transplant demonstrated a preserved cellular response to the influenza vaccine^20,21^. An additional assessment of cell-mediated vaccine responses, such as testing of T-cell activation to influenza antigens, in addition to assessment of humoral response may produce a more complete picture of vaccine response in our population. Our study is further limited by the single centre open-label design with a timeline overlapping the COVID-19 pandemic which resulted in strict restrictions on travel in Australia, including complete closure of both the international and Western Australian interstate border. This resulted in low circulating levels of influenza locally and nationally^22,23^, with no cases of clinical influenza illness identified in our cohort. As such clinical measures of vaccine efficacy could not be determined, however, given that higher antibody levels are associated with a reduced risk of infection, immunogenicity is considered an acceptable surrogate clinical endpoint for vaccine studies^24^. In addition, the number of patients recruited limited our assessment of clinical variables that may help predict antibody response, necessitating larger studies to elucidate features associated with immunogenicity. Finally, whilst we have demonstrated benefit of an additional influenza vaccine dose in this immunocompromised patient population, the humoral immune response remains inferior compared to healthy children receiving standard quadrivalent schedule^25–27^, indicating that further strategies are required to maximise protection against influenza for children with cancer.

In summary, we have conducted the first modern clinical study investigating a boosted influenza vaccination schedule for children undergoing treatment for cancer. Our study demonstrated that an additional vaccine dose was safe, feasible and led to a significant increase in influenza antibody titres. Overall, these findings provide a practical and readily implementable strategy to help protect immunocompromised children undergoing treatment for cancer from influenza.

## Methods

### Trial design and participants

We conducted a prospective single-arm open-label study recruiting children from 6 months to 18 years of age who were actively receiving cancer therapy at Perth Children’s Hospital, Western Australia during the influenza seasons of 2020 and 2021 (March–September). The study was approved by the Child and Adolescent Health Service Ethics Committee (RGS0000003544) and registered on the Australian and New Zealand Clinical Trials Registry (ACTRN12620000548932). Written informed consent was obtained from the parents and/or legal guardians of each child prior to enrollment and assent was obtained from the participant where appropriate. Patient data was collected and stored securely in REDCap with access given to approved research investigators.

### Exclusion criteria

Exclusion criteria included anaphylaxis to previous doses of any influenza vaccines, history of Guillain-Barré syndrome post-influenza vaccine, receipt of immunoglobulin within the last 3 months prior to vaccination and receipt of an autologous or allogeneic haematopoietic stem cell transplantation. Patients who received any prior immunotherapy (e.g., blinatumomab, chimeric antigen receptor T-cell therapy) were excluded from this primary analysis.

### Vaccine and study procedures

Patients were vaccinated with a 0.5 ml dose of the quadrivalent inactivated influenza vaccine FluQuadri (Sanofi-Aventis, Australia). Children <9 years of age were given three doses of the vaccine and those ≥9 years of age were given two doses, at least 4 weeks apart, instead of the usual two and one dose schedules respectively. This schedule was administered to all participants regardless of prior vaccination history. The specific strains included in the vaccine were A/Brisbane/02/2018 (H1N1), A/South Australia/34/2019 (H3N2), B/Washington/02/2019 and B/Phuket/3073/2013 for 2020; and A/Victoria/2570/2019 (H1N1), A/Hong Kong/2671/2019 (H3N2), B/Washington/02/2019 and B/Phuket/3073/2013 for 2021. Vaccinations were administered by intramuscular injection by oncology research nurses during an inpatient ward admission or outpatient clinic visit to the Department of Clinical Haematology, Oncology, Blood and Marrow Transplantation at Perth Children’s Hospital.

### Safety evaluations

To assess safety of the boosted influenza vaccine schedule as a primary study aim, children were observed for 30 min post-vaccination and parents were interviewed 7 days post-vaccination to identify any adverse events following immunisation (AEFIs). Intensity of AEFIs were categorised and graded (mild, moderate or severe) and attribution of the influenza vaccine to AEFIs was defined, as detailed in Supplementary Tables 3–5. To ensure the safety of all study participants, a Data Safety Monitoring Board (DSMB) comprising of six paediatric experts in haematology, oncology, infectious disease, immunology, neurology and biostatistics was appointed. Any unexpected, severe or serious AEFIs were to be reported to the DSMB and relevant state and national authorities within 24 h. Serious AEFIs that required expedited reporting are specified in Supplementary Table 6. Stopping rules comprised of severe or unexpected AEFIs that were similar in nature and could not be attributable to any other cause in two or more subjects receiving the influenza vaccine or a serious AEFI in one subject receiving the influenza vaccine, which would trigger an immediate investigation by the DSMB.

### Immunogenicity assays

Blood samples were taken at study entry to measure baseline total lymphocyte count, lymphocyte subsets and immunoglobulin levels. Blood was taken prior to each vaccination and 4 weeks following the final vaccination to assess influenza-specific immune responses. Following collection, samples were centrifuged at 2000 RPM for 25 min at room temperature and sera stored at −20 °C. At the end of the study, samples were sent to the World Health Organization (WHO) Collaborating Centre for Reference and Research on Influenza, Victorian Infectious Diseases Reference Laboratory in Melbourne where HI assays were performed to determine specific influenza antibody titres towards each virus in the vaccine, as previously described^28^. Assessment was made using influenza antigens propagated in both embryonated hen’s eggs and mammalian cells, where the egg-grown antigen provides an indication of response to the vaccine, while cell-grown antigen provides an indication of protection against circulating viruses^29^. In brief, sera were treated with receptor destroying enzyme (Denka Seiken) then adsorbed with a mix of erythrocytes from guinea pigs and turkeys to remove non-specific agglutinins. Sera were diluted two-fold starting at 1:10 to a maximum dilution of 1:10,240 then incubated with virus at 4 hemagglutination units/25ul before adding 1% erythrocytes. For A/H3N2 viruses, assays were performed in U bottom plates and read using an automated hemagglutination analyser (CypherOne, InDevR) after 90 min of incubation with guinea pig erythrocytes. For A/H1N1 and B viruses, assays were performed in V bottom plates and read after 30 min of incubation with turkey erythrocytes as the reciprocal of the highest serum dilution causing complete inhibition of agglutination when plates are tilted.

### Statistical analysis

The primary efficacy aim of our study was to determine whether a boosted influenza vaccine schedule could improve immunogenicity in children receiving treatment for cancer. For each strain, differences between the immune responses elicited by the boosted schedule and the standard schedule were summarised by their respective GMTs, with the relative immune response assessed by application of a paired-sample *t*-test of the log-transformed post-vaccination titres. Construction of 95% CIs for GMT summary statistics utilised a log-normal approximation for the distribution of antibody levels pre- and post-vaccination. Seroconversion was defined as either a fourfold increase in HI antibody titre if the pre-vaccination titre was ≥10 or a rise in HI titre from <10 to ≥40 following vaccination^30^. Comparison with the standard influenza vaccine schedule included use of a McNemar test to assess the difference in percentage of patients who seroconverted following the penultimate (standard schedule) and final (boosted schedule) dose of the vaccine. Univariate logistic regression analysis was used to identify factors associated with HI antibody titres and response. Data were analysed using R Statistical Software (4.4.1; R Core Team, 2024) within the RStudio integrated development environment (RStudio Team, Boston MA).

## Supplementary information

Supplementary Information

Research Protocol

## Data Availability

The raw data generated from this study can be made available upon reasonable request.
