# Supplementary material for: Boosting the influenza vaccine schedule in children with cancer: a prospective open-label study
Source: NPJ Vaccines. 2025 Aug 26;10:203. doi: 10.1038/s41541-025-01256-0 (PMC12379222; doi:10.1038/s41541-025-01256-0)
Supplement: Supplementary file 1 — Supplementary Information [file 41541_2025_1256_MOESM1_ESM.pdf]

Supplementary Information

Boosting the influenza vaccine schedule in children with cancer: a prospective open-label study.

Sung K Chiu, Eliska Furlong, Elizabeth J McKinnon, Annette Fox, Stephany Sánchez Ovando, Louise Carolan, Andrew McLean-Tooke, Joyce Oommen, Daniel K Yeoh, Laurence C Cheung, Nicholas G Gottardo, & Rishi S Kotecha

Table of Contents

SUPPLEMENTAL TABLE 1. CLASSIFICATION OF TREATMENT ACCORDING TO INTENSITY. 3

SUPPLEMENTAL TABLE 2. UNIVARIATE LOGISTIC REGRESSION ANALYSIS TO IDENTIFY FACTORS ASSOCIATED WITH ANTIBODY TITRES AND TITRE RISES AGAINST EACH INFLUENZA VACCINE ANTIGEN. ..4

SUPPLEMENTAL TABLE 3. INTENSITY GRADING OF ADVERSE EVENTS FOLLOWING IMMUNISATION (AEFIS). 6

SUPPLEMENTAL TABLE 4. DEFINITION OF ADVERSE EVENTS FOLLOWING IMMUNISATION (AEFIS) IN RELATION TO CAUSALITY. 6

SUPPLEMENTAL TABLE 5. CATEGORIES AND GRADING DEFINITION OF ADVERSE EVENTS FOLLOWING IMMUNISATION..........7

SUPPLEMENTAL TABLE 6. SERIOUS ADVERSE EVENTS FOLLOWING IMMUNISATION THAT REQUIRED REPORTING TO THE DATA SAFETY MONITORING BOARD. 9

SUPPLEMENTAL FIGURE 1. CONSORT DIAGRAM OF STUDY POPULATION. 11

SUPPLEMENTAL FIGURE 2. CORRELATION BETWEEN EGG-GROWN AND CELL-GROWN HEMAGGLUTINATION INHIBITION ANTIBODY TITRES..........12

Supplemental Table 1. Classification of treatment according to intensity.

<table>
<tr><th colspan="2">Tumour type</th><th>High intensity</th><th>Low intensity</th></tr>
<tr><td colspan="2">Acute Lymphoblastic Leukaemia/Lymphoblastic Lymphoma</td><td>Induction
Augmented Consolidation
Delayed Intensification</td><td>Standard Consolidation
Interim Maintenance
Maintenance</td></tr>
<tr><td colspan="2">Acute Myeloid Leukaemia</td><td>All therapy except Maintenance for acute promyelocytic leukaemia (APML)</td><td>Maintenance for APML</td></tr>
<tr><td colspan="2">Non-Hodgkin Lymphoma</td><td>Cytarabine/Methotrexate (CYM)</td><td>Doxorubicin/Mercaptopurine/Prednisone</td></tr>
<tr><td colspan="2">Hodgkin Lymphoma</td><td>Doxorubicin/Bleomycin/Vincristine/Etoposide – Prednisone/Cyclophosphamide (ABVE-PC)</td><td></td></tr>
<tr><td colspan="2">Langerhans Cell Histiocytosis</td><td></td><td>Vinblastine/Prednisone</td></tr>
<tr><td rowspan="4">Central Nervous System Tumours</td><td>Medulloblastoma</td><td>Lomustine/Cisplatin/Vincristine
Cyclophosphamide/Vincristine</td><td></td></tr>
<tr><td>Ependymoma</td><td>Vincristine/Carboplatin/Cyclophosphamide/Etoposide</td><td></td></tr>
<tr><td>High grade glioma</td><td>Cyclophosphamide/Carboplatin/Etoposide</td><td>Temozolomide</td></tr>
<tr><td>Low grade glioma</td><td></td><td>Vinblastine
Bevacizumab/Irinotecan
Carboplatin/Vincristine</td></tr>
<tr><td colspan="2">Wilms Tumour</td><td></td><td>Vincristine/Actinomycin D
Vincristine/Actinomycin D/Doxorubicin</td></tr>
<tr><td colspan="2">Ewing Sarcoma</td><td>Vincristine/Ifosfamide/Doxorubicin/Etoposide (VIDE)
Vincristine/Doxorubicin/Cyclophosphamide – Ifosfamide/Etoposide (VDC-IE)</td><td></td></tr>
<tr><td colspan="2">Rhabdomyosarcoma</td><td>Vincristine/Actinomycin D/Cyclophosphamide (VAC)</td><td>Vincristine/Irinotecan (VI)</td></tr>
<tr><td colspan="2">Retinoblastoma</td><td></td><td>Carboplatin/Etoposide/Vincristine (CEV)</td></tr>
<tr><td colspan="2">Germ Cell/
Sex Cord Stromal Tumour</td><td>Bleomycin/Etoposide/Cisplatin (BEP)</td><td></td></tr>
<tr><td colspan="2">Nasopharyngeal Carcinoma</td><td>Cisplatin/5-Fluorouracil</td><td></td></tr>
</table>

Supplemental Table 2. Univariate logistic regression analysis to identify factors associated with antibody titres and titre rises against each influenza vaccine antigen. GMT, Geometric mean titre; GMR, Geometric mean ratio.

A/H1N1	Baseline		End of study			
**Group**	**GMT**	**P**	**GMT**	**P**	**GMR**	**P**
Age < 9 years	11 (7,17)	<0·001	50 (25,101)	0·002	4·63 (2·66,8·08)	0·54
Age ≥ 9 years	56 (34,93)		203 (115,359)		3·64 (2·01,6·58)	
Female	19 (10,37)	0·50	113 (43,298)	0·69	5·88 (2·78,12·44)	0·25
Male	25 (15,42)		91 (52,160)		3·58 (2·22,5·77)	
Solid tumour	23 (12,47)	>0·99	110 (55,217)	0·68	4·68 (2·50,8·78)	0·63
Haematological malignancy	23 (14,39)		90 (47,174)		3·86 (2·29,6·52)	
Non-intensive treatment	19 (8,45)	0·56	59 (24,143)	0·20	3·08 (1·55,6·13)	0·33
Intensive treatment	25 (16,40)		115 (65,204)		4·58 (2·82,7·45)	
Previously unvaccinated	25 (14,46)	0·76	110 (44,276)	0·69	4·36 (2·07,9·18)	0·84
Previously vaccinated	22 (13,38)		89 (51,155)		4·00 (2·50,6·41)	
Lymphocytes < normal range	25 (16,39)	0·56	87 (52,145)	0·43	3·53 (2·31,5·39)	0·15
Lymphocytes ≥ normal range	19 (8,44)		151 (38,596)		8·00 (2·68,23·91)	
IgG < normal range	19 (10,39)	0·48	57 (22,152)	0·13	2·96 (1·41,6·23)	0·22
IgG ≥ normal range	26 (16,43)		132 (79,219)		5·04 (3·17,8·02)	

A/H3N2	Baseline		End of study			
**Group**	**GMT**	**P**	**GMT**	**P**	**GMR**	**P**
Age < 9 years	25 (18,34)	0·03	80 (46,140)	0·17	3·22 (1·98,5·23)	0·99
Age ≥ 9 years	42 (29,60)		141 (76,263)		3·20 (1·82,5·64)	
Female	21 (14,31)	0·02	71 (31,164)	0·25	3·40 (1·54,7·50)	0·85
Male	38 (28,50)		122 (75,197)		3·14 (2·08,4·75)	
Solid tumour	31 (20,48)	0·88	145 (75,279)	0·22	4·42 (2·46,7·92)	0·18
Haematological malignancy	32 (24,44)		87 (51,149)		2·71 (1·70,4·30)	
Non-intensive treatment	32 (20,52)	0·96	87 (36,211)	0·61	2·71 (1·19,6·15)	0·60
Intensive treatment	32 (24,42)		111 (69,180)		3·42 (2·27,5·14)	
Previously unvaccinated	25 (18,36)	0·13	128 (61,271)	0·46	4·83 (2·48,9·40)	0·10
Previously vaccinated	37 (26,51)		93 (56,153)		2·54 (1·66,3·86)	
Lymphocytes < normal range	33 (25,43)	0·61	95 (60,150)	0·39	2·83 (1·92,4·16)	0·22
Lymphocytes ≥ normal range	28 (16,49)		151 (54,426)		5·34 (1·96,14·54)	
IgG < normal range	27 (18,40)	0·29	78 (40,150)	0·25	2·87 (1·61,5·12)	0·62
IgG ≥ normal range	35 (26,48)		125 (73,215)		3·44 (2·13,5·56)	

Supplemental Table 2.

B/Washington	Baseline		End of study			
Group	GMT	P	GMT	P	GMR	P
Age < 9 years	51 (38,70)	<0·001	119 (83,171)	0·003	2·32 (1·61,3·33)	0·91
Age ≥ 9 years	126 (91,174)		284 (183,441)		2·25 (1·61,3·16)	
Female	71 (43,118)	0·64	244 (134,447)	0·20	3·43 (2·15,5·47)	0·04
Male	81 (61,109)		157 (112,222)		1·94 (1·46,2·56)	
Solid tumour	106 (77,147)	0·04	291 (184,460)	0·01	2·74 (1·90,3·94)	0·24
Haematological malignancy	66 (47,93)		137 (95,198)		2·07 (1·50,2·87)	
Non-intensive treatment	52 (28,96)	0·09	113 (60,213)	0·08	2·18 (1·32,3·60)	0·82
Intensive treatment	90 (70,117)		210 (151,292)		2·33 (1·74,3·10)	
Previously unvaccinated	73 (50,107)	0·67	155 (100,242)	0·44	2·12 (1·57,2·85)	0·59
Previously vaccinated	81 (58,114)		196 (131,293)		2·40 (1·68,3·43)	
Lymphocytes < normal range	71 (53,94)	0·03	171 (122,241)	0·53	2·43 (1·85,3·18)	0·33
Lymphocytes ≥ normal range	120 (81,178)		214 (110,414)		1·78 (0·96,3·31)	
IgG < normal range	59 (39,90)	0·09	96 (58,159)	0·001	1·62 (1·06,2·48)	0·03
IgG ≥ normal range	92 (68,125)		259 (187,357)		2·80 (2·11,3·73)	

B/Phuket	Baseline		End of study			
Group	GMT	P	GMT	P	GMR	P
Age < 9 years	117 (92,148)	0·003	202 (144,282)	0·02	1·73 (1·21,2·45)	0·56
Age ≥ 9 years	189 (152,235)		378 (248,576)		2·00 (1·37,2·92)	
Female	160 (123,208)	0·45	308 (191,498)	0·52	1·92 (1·29,2·87)	0·82
Male	141 (113,176)		257 (183,359)		1·82 (1·32,2·51)	
Solid tumour	165 (130,210)	0·26	425 (263,687)	0·02	2·57 (1·67,3·96)	0·05
Haematological malignancy	137 (108,173)		211 (154,289)		1·54 (1·13,2·10)	
Non-intensive treatment	108 (69,169)	0·09	174 (108,283)	0·04	1·61 (0·94,2·75)	0·53
Intensive treatment	162 (137,193)		315 (229,434)		1·94 (1·45,2·60)	
Previously unvaccinated	113 (86,149)	0·02	261 (174,394)	0·84	2·31 (1·57,3·40)	0·15
Previously vaccinated	172 (139,212)		277 (191,401)		1·61 (1·15,2·25)	
Lymphocytes < normal range	143 (118,174)	0·61	279 (203,383)	0·60	1·95 (1·46,2·58)	0·41
Lymphocytes ≥ normal range	160 (105,243)		240 (144,400)		1·50 (0·82,2·75)	
IgG < normal range	122 (91,164)	0·11	165 (104,260)	0·005	1·35 (0·85,2·14)	0·07
IgG ≥ normal range	163 (132,201)		362 (265,496)		2·23 (1·66,2·99)	

Supplemental Table 3. Intensity grading of adverse events following immunisation (AEFIs).

**Intensity of AEFIs**	**Definition**
Mild	Does not interfere with subject's usual function.
Moderate	Interferes to some extent with subject's usual function.
Severe	Interferes significantly with subject's usual function.

Supplemental Table 4. Definition of adverse events following immunisation (AEFIs) in relation to causality.

**Causality of AEFIs**	**Definition**
Related event	There is a plausible temporal relationship between the onset of the adverse event (AE) and administration of the influenza vaccine, and the AE cannot be readily explained by the subject's clinical state, intercurrent illness or concomitant therapies.
Unrelated event	Evidence exists that the AE has aetiology other than the influenza vaccine and/or the AE has no plausible temporal relationship to the influenza vaccine administration.
Expected event	An AE that is commonly reported by drug trial participants and listed in the product information brochure.
Unexpected event	An unexpected event is an AE not listed in the product information brochure. This includes AEs for which the specificity or severity is not consistent with the description in the product information brochure.

Supplemental Table 5. Categories and grading definition of adverse events following immunisation.

Type of reaction	Severity	Definition
Local		
Tenderness	Mild	Hurts if gently touched (subject whimpers, winces, protests or withdraws)
	Moderate	Hurts if gently touched with crying
	Severe	Pain persisting for more than three days post immunisation
Erythema	Mild	0·5 to 2·0 cm
	Moderate	>2·0 cm to 7·0 cm
	Severe	>7·0 cm
Swelling/induration	Mild	≤1·0 cm
	Moderate	>1·0 to 5·0 cm
	Severe	>5·0 cm
Systemic		
Fever	Mild	38·0 to 39·0 °C
	Moderate	>39·0 - 40·0 °C
	Severe	>40·0 °C
Irritability	Mild	Easily consolable
	Moderate	Requiring increased attention
	Severe	Crying which is continuous and unaltered for longer than three hours
Drowsiness	Mild	Increased or prolonged sleeping bouts
	Moderate	Slightly subdued interfering with daily activity
	Severe	Requiring medical investigation or hospitalisation
Decreased appetite	Mild	Decreased interest in eating
	Moderate	Decreased oral intake
	Severe	Minimal oral intake requiring medical intervention for nutritional support
Vomiting	Mild	Easily tolerated by the participant, causing minimal discomfort and does not interfere with everyday activities
	Moderate	Sufficiently discomforting to interfere with normal everyday activities
	Severe	Persistent vomiting requiring medical intervention

Diarrhoea	Mild	Easily tolerated by the participant, causing minimal discomfort and does not interfere with everyday activities
	Moderate	Sufficiently discomforting to interfere with normal everyday activities
	Severe	Persistent diarrhoea requiring medical intervention
Myalgia	Mild	Mild muscle pain
	Moderate	Moderate muscle pain, limiting instrumental activities of daily living but not self-care
	Severe	Severe muscle pain, limiting instrumental activities of daily living and self-care
Headache	Mild	Mild headache
	Moderate	Moderate headache, limiting instrumental activities of daily living but not self-care
	Severe	Severe headache, limiting activities of daily living and self-care

Supplemental Table 6. Serious adverse events following immunisation that required reporting to the data safety monitoring board.

**Abscess**	Occurrence of a fluctuant or draining fluid-filled lesion at the site of injection, with or without fever.
**Acute flaccid paralysis**	Acute onset of flaccid paralysis of one or more limbs following any vaccine.
**Generalised allergic reaction**	A non-anaphylactic, generalised reaction characterised by one or more symptoms or signs of skin and/or gastrointestinal tract involvement WITHOUT respiratory or cardiovascular involvement.
**Anaphylaxis**	A rapidly evolving generalised multi-system allergic reaction characterised by one or more symptoms or signs of respiratory and/or cardiovascular involvement AND involvement of other systems such as the skin or gastrointestinal tract.
**Arthralgia**	Joint pain without redness or swelling.
**Arthritis**	Joint pain with redness and/or swelling.
**Brachial neuritis**	Pain in arm causing persisting weakness of limb on side of vaccination.
**Death**	Any death of a vaccine recipient temporally linked to vaccination, where no other clear cause of death can be established.
**Encephalopathy**	An acute onset of major neurological illness temporally linked with vaccination and characterised by any two or more of the following three conditions: seizures, severe alteration in level of consciousness or mental status (behaviour and/or personality) lasting for one day or more, and/or focal neurological signs which persist for one day or more.
**Encephalitis**	Characterised by the above-mentioned symptoms and signs of cerebral inflammation and, in many cases, cerebrospinal fluid (CSF) pleocytosis.
**Extensive limb swelling**	With or without redness which extends from the joint above to the joint below the injection site, or beyond a joint (above or below the injection site), or results in the circumference of the limb being twice the normal size.
**Guillain-Barré syndrome**	Acute onset of rapidly progressive, ascending, symmetrical flaccid paralysis, without fever at onset of paralysis and with or without sensory loss. Diagnosed by CSF investigation showing dissociation between cellular count and protein content.
**Hypotonic–hyporesponsive episode (shock, collapse)**	The sudden onset of pallor or cyanosis, limpness (muscle hypotonia), and reduced responsiveness or unresponsiveness occurring after vaccination, where no other cause is evident such as a vasovagal episode or anaphylaxis. The episode usually occurs one to 48 hours after vaccination and resolves spontaneously.
**Severe injection site reaction**	Reaction (redness and/or swelling) at site of injection which persists for more than three days AND is associated with ongoing symptoms such as pain or an inability to use the limb (see 'Brachial neuritis' above), and does not fulfil the case definition for extensive limb swelling and requires hospitalisation.
**Intussusception**	The invagination of a proximal segment of bowel into the distal bowel lumen.

**Lymphadenitis**	Occurrence of either at least one lymph node, 1.5 cm in diameter or larger, or a draining sinus over a lymph node.
**Meningitis**	Acute onset of major illness with fever and often neck stiffness/positive meningeal signs (Kernig, Brudzinski) and with CSF pleocytosis.
**Nodule**	A discrete or well demarcated soft tissue mass or lump that is firm and is at the injection site in the absence of abscess formation, warmth and erythema.
**Orchitis**	Swelling with pain and/or tenderness of testes.
**Osteitis**	Inflammation of the bone due to vaccination.
**Osteomyelitis**	Proven bacterial infection of bone.
**Parotitis**	Swelling and/or tenderness of parotid gland or glands.
**Rash**	Severe or unusual rash.
**Seizure**	Witnessed sudden loss of consciousness and generalised, tonic, clonic, tonic-clonic, or atonic motor manifestations.<br>• febrile seizures: with fever ≥38.5°C,<br>• afebrile seizures: without fever,<br>• syncopal seizures: syncope/vasovagal episode followed by seizure(s).
**Thrombocytopenia**	Platelet count <50 x 10^9/L.
**Toxic shock syndrome**	Abrupt onset of fever, vomiting, watery diarrhoea and shock within a few hours of vaccination which can be associated with other conditions listed here.
**Other serious events**	Any unusual event that does not fit into any of the categories listed above, but is of medical or epidemiological interest.

Supplemental Figure 1. Consort diagram of study population.

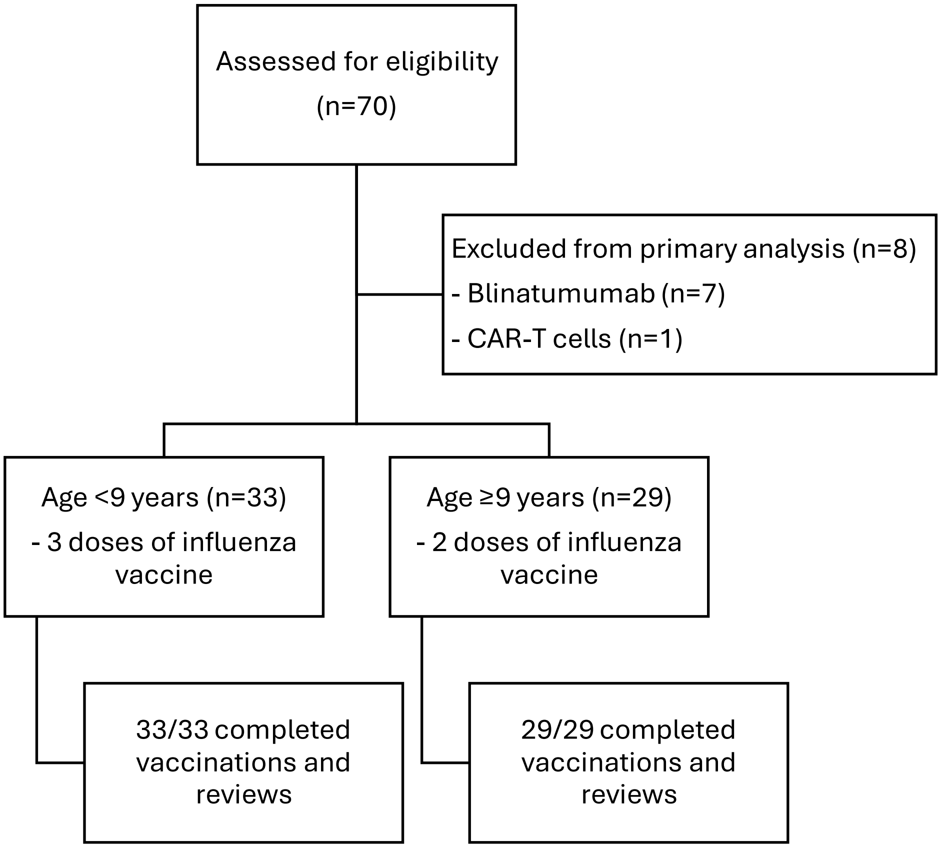

Supplemental Figure 2. Correlation between egg-grown and cell-grown hemagglutination inhibition antibody titres.

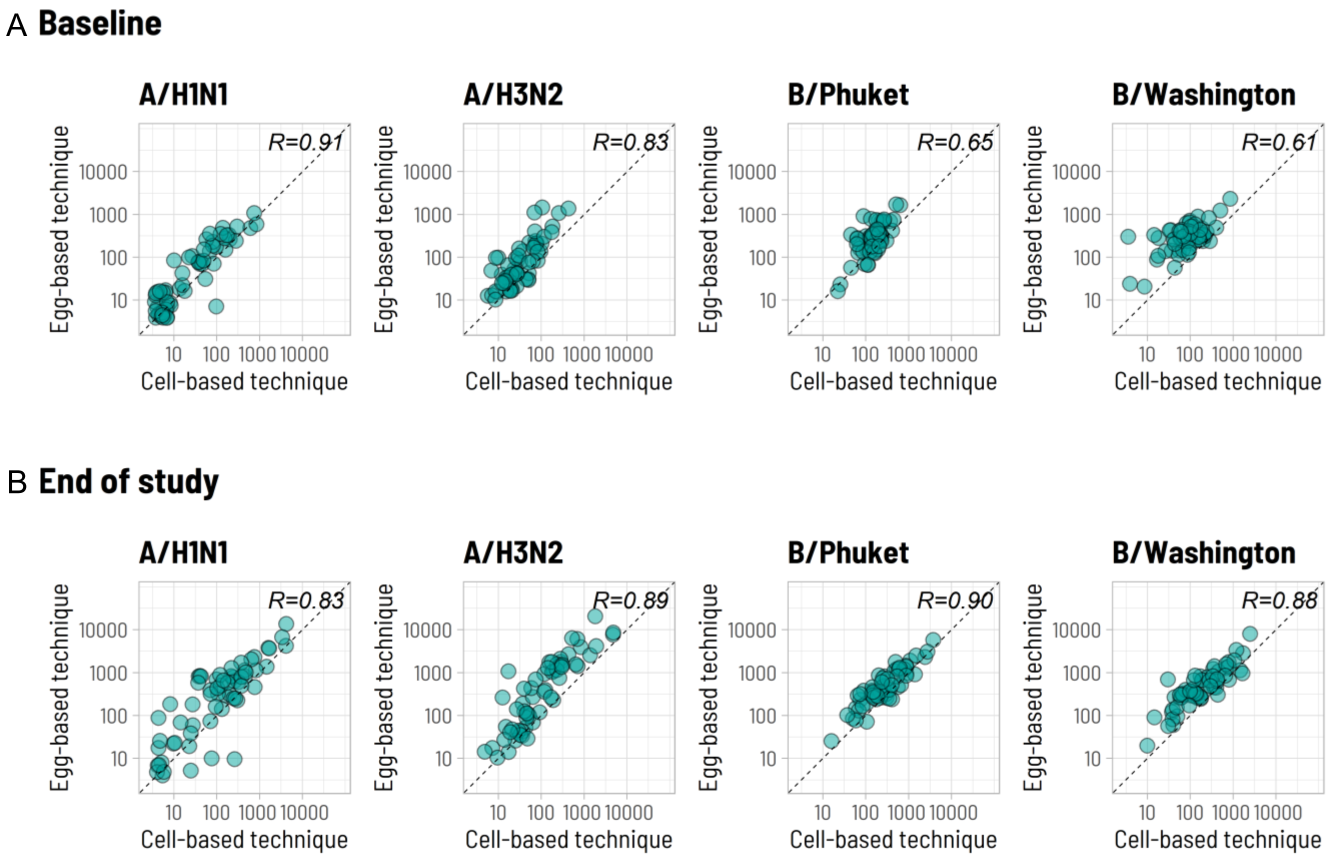

R is the Spearman correlation coefficient.
