# Supplementary material for: Boosting the influenza vaccine schedule in children with cancer: a prospective open-label study
Source: NPJ Vaccines. 2025 Aug 26;10:203. doi: 10.1038/s41541-025-01256-0 (PMC12379222; doi:10.1038/s41541-025-01256-0)
Supplement: Supplementary file 2 — Research Protocol [file 41541_2025_1256_MOESM2_ESM.pdf]

Government of Western Australia
Child and Adolescent Health Service

Perth Children's Hospital

TELETHON KIDS INSTITUTE
Discover. Prevent. Cure.

Evaluation of an enhanced influenza vaccination schedule in immunocompromised children undergoing treatment for cancer by assessment of cellular and humoral response

Flu Study 2020

Version date: 27 Nov 2019
Protocol version: 2

Project start date: 2 Feb 2020
Project finish date: 31 Jan 2022

TABLE OF CONTENTS

GLOSSARY OF ABBREVIATIONS

ABSTRACT

PROJECT DESIGN SCHEMA

1.0 BACKGROUND AND STUDY RATIONALE
- 1.1 Influenza infection in paediatric patients undergoing treatment for cancer
- 1.2 Current influenza vaccination schedule in children undergoing treatment for cancer
- 1.3 Standard evaluation of influenza vaccine response
- 1.4 Enhanced influenza vaccination schedule and extended vaccine response evaluation
- 1.5 Summary

2.0 STUDY OBJECTIVES
- 2.1 Safety objectives
- 2.2 Efficacy objectives
  - 2.2.1 Primary efficacy objectives
  - 2.2.2 Secondary efficacy objectives
- 2.3 Exploratory objectives

3.0 STUDY ENROLMENT
- 3.1 Participant enrolment
- 3.2 Participant/Parent Information sheet and Consent form
- 3.3 Participant recruitment
- 3.4 Participant registration
- 3.5 Timing
- 3.6 Participant withdrawal criteria
- 3.7 Number of participants
- 3.8 Duration of study

4.0 PARTICIPANT ELIGIBILITY CRITERIA
- 4.1 Inclusion criteria
- 4.2 Exclusion criteria

5.0 STUDY DESIGN
- 5.1 Type of study
- 5.2 Study interventions
- 5.3 Study visit schedule
  - 5.3.1 Visit 1
  - 5.3.2 Visit 2
  - 5.3.3 Visit 3
  - 5.3.4 Visit 4

6.0 VACCINE
6.1 Influenza vaccine
6.2 Contraindications and precautions

7.0 LABORATORY EVALUATIONS
7.1 Assessment of humoral response to influenza vaccine
7.2 Assessment of cellular response to influenza vaccine
7.3 Laboratory testing for influenza infection

8.0 SAFETY, ADVERSE EVENT MONITORING AND REPORTING
8.1 Adverse events following immunisation (AEFIs)
8.1.1 Definition and Grading of AEFIs
8.1.2 Relationship of AEFIs to the influenza vaccine
8.1.3 Definition of a mild and moderate AEFI
8.1.4 Definition of a serious AEFI
8.2 AEFI(s) Evaluation Timeline
8.3 Reporting and monitoring of AEFIs
8.3.1 Reporting responsibilities
8.3.2 Required reporting criteria
8.3.3 Reporting Timelines
8.3.4 Data Safety Monitoring Board (DSMB)
8.4 Stopping rules

9.0 STATISTICS
9.1 Statistical design
9.2 Sample size estimation
9.3 Statistical analysis

10.0 RECORD AND SAMPLE MANAGEMENT
10.1 Record keeping and handling
10.2 Samples collection and shipping

11.0 QUALITY CONTROL AND QUALITY ASSURANCE

12.0 ETHICAL AND REGULATORY CONSIDERATIONS
12.1 Ethics
12.2 Project monitoring

APPENDIX

REFERENCES

GLOSSARY OF ABBREVIATIONS

**ADL**	Activities of daily living
**AE**	Adverse event
**AEFI**	Adverse event following immunisation
**AEFI-CAN**	Adverse Events Following Immunisation - Clinical Assessment Network
**AI**	Associate Investigator
**AIVC**	Australian Influenza Vaccine Committee
**ANC**	Absolute neutrophil count
**ATAGI**	Australian Technical Advisory Group on Immunisation
**CPI**	Coordinating Principal Investigator
**CPMP**	Committee for Proprietary Medicinal Products
**CTCAE**	Common Terminology Criteria for Adverse Events
**CTLA-4**	Cytotoxic T-lymphocyte associated protein 4
**CVAD**	Central venous access device
**DSMB**	Data Safety Monitoring Board
**GCP**	Good Clinical Practice
**GISN**	Global Influenza Surveillance Network
**GMFI**	Geometric mean fold increase
**HHI**	Hypotonic-hyporesponsive episode
**HI**	Influenza-specific hemagglutinin inhibition
**HOPAG**	Haematology and Oncology Parent Advisory Group
**HREC**	Human Research Ethics Committee
**ILI**	Influenza like illness
**NCIRS**	National Centre of Immunisation Research and Surveillance
**NHMRC**	National Health and Medical Research Council
**PCH**	Perth Children’s Hospital
**PCR**	Polymerase chain reaction
**PD-1**	Programmed cell death protein 1
**PD-L1**	Programmed cell death protein ligand 1
**RGO**	Research Governance Office
**RGS**	Research Governance Service
**SAE**	Severe Adverse Event
**SASC**	Scientific Advisory Sub-Committee
**TGA**	Therapeutic Goods Administration
**TKI**	Telethon Kids Institute
**VIDRL**	Victorian Infectious Diseases Reference Laboratory
**WACPCN**	WA Cancer and Palliative Care Network
**WAVSS**	WA Vaccine Safety Surveillance
**WHO**	World Health Organization

ABSTRACT

Influenza is a common seasonal infection associated with significant morbidity, mortality and prolonged hospitalisations among children undergoing treatment for cancer. Currently, all children in Australia aged 6 months to 18 years undergoing immunosuppressive therapy, such as treatment for cancer, are recommended to receive annual influenza vaccination.

The current immunisation schedule is supported by scientific evidence demonstrating safety, efficacy and clinical benefit of the inactivated influenza vaccine in immunocompromised children undergoing treatment for cancer. However, immunogenicity is reduced compared to the healthy population. Studies in immunosuppressed adults undergoing treatment for haematological malignancies suggest enhanced response to the influenza vaccine when given repeatedly.

In this study we propose that the inactivated influenza vaccine response in children and adolescents who are being treated for cancer could be improved by introducing an additional vaccine dose to the current one-dose schedule in patients ≥9 years of age, and the two-dose regimen in patients <9 years of age. We also propose that developing an additional laboratory method of assessing the cellular immune response to the influenza vaccine could be of benefit, given the limitations of the standardised antibody response in immunosuppressed populations. In addition, the study design will allow assessment of predictors of influenza vaccine immune response with the aim of optimising timing of the vaccination.

The safety of an enhanced influenza vaccine schedule in children undergoing treatment for cancer will be closely monitored by an independent advisory committee composed of several specialists from related paediatric disciplines. Participants in the <9 years of age group will be informed about the novelty of the approach and need for enhanced monitoring. Participants aged 6 to 35 months will only receive an additional influenza vaccine if an initial safety run in of the enhanced schedule conducted in children 3 to <9 years of age is determined to be safe and tolerable.

It is estimated that 80 subjects will be recruited over the study period. This is based on the incidence and prevalence of patients diagnosed and receiving treatment for cancer at Perth Children's Hospital Haematology and Oncology Department.

Overall, we aim to establish an evidence-based influenza vaccine immunisation schedule for immunocompromised children and adolescents undergoing treatment for cancer in Western Australia. We also aim to develop and validate a complementary assay for monitoring vaccine response in immunocompromised children. Ultimately, we envisage positive outcomes to have a global impact, with the potential for recommendations from this project to be implemented into national and international immunisation guidelines.

PROJECT DESIGN SCHEMA

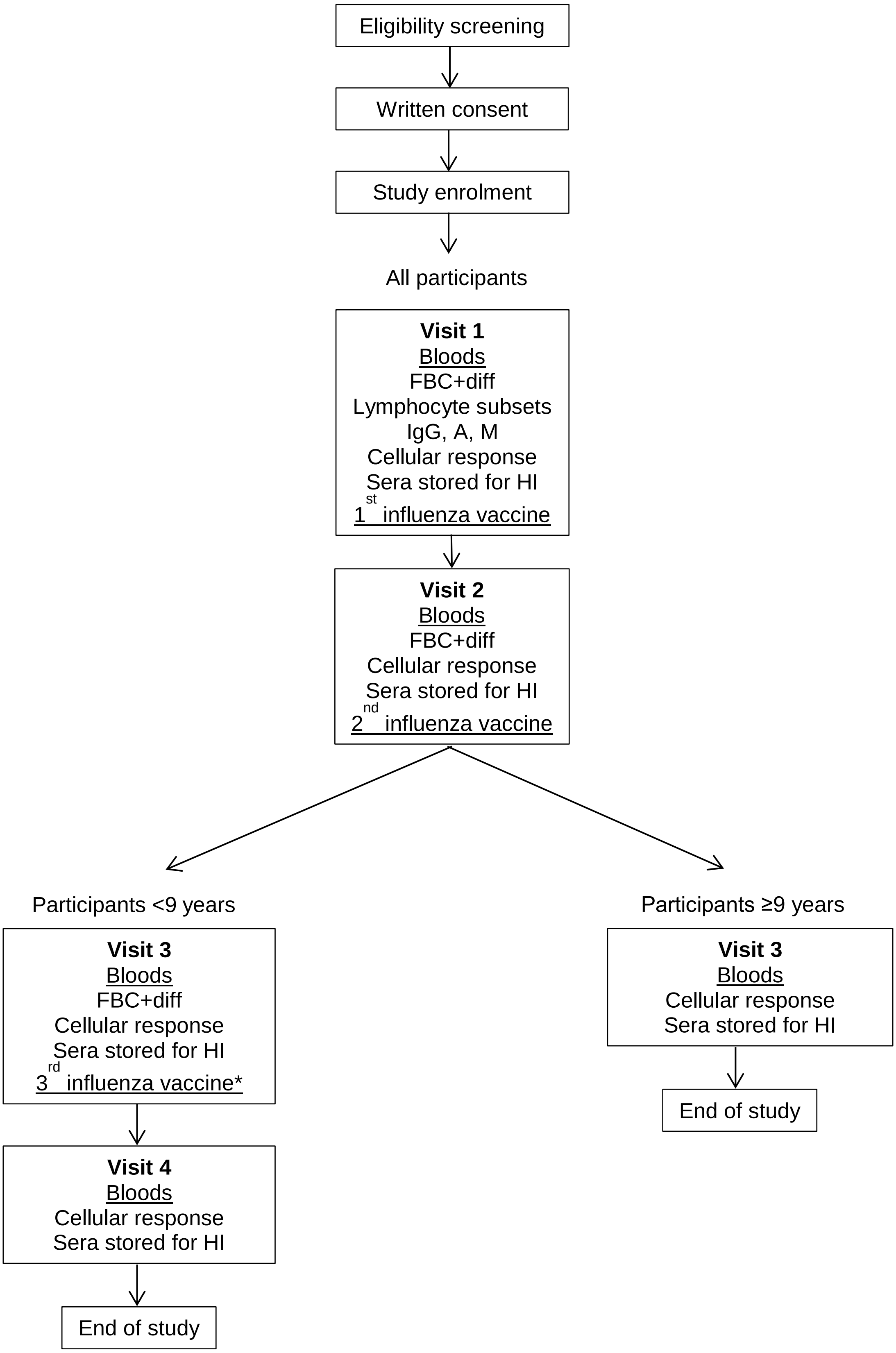

*****children aged 6 to 35 months will only receive the 3rd influenza vaccine dose if an initial safety run in phase determines that the third dose of the vaccine is safe and tolerable in children aged 3 to <9 years of age

1.0 BACKGROUND AND STUDY RATIONALE

1.1 Influenza infection in paediatric patients undergoing treatment for cancer

Remarkable progress has been made in the treatment of childhood cancer over the last 40 years, with a significant reduction in overall mortality in developed countries (1, 2). The success of cancer treatment can be largely attributable to intensified and prolonged chemotherapy. However, this has consequently resulted in a greater burden of complications related to increased toxicity and prolonged periods of immunosuppression.

Influenza is a common seasonal infection associated with significant morbidity, mortality and prolonged hospitalisations among children undergoing treatment for cancer due to chemotherapy induced immunosuppression (3, 4). These patients are not only more susceptible to the influenza virus when compared to healthy children but often suffer from a prolonged and more severe course of illness, ultimately resulting in delayed chemotherapy (5, 6). Associated co-morbidities, such as bacterial infections, have been reported in up to 15% of patients suffering from an influenza related illness, further complicating the course of recovery (7).

1.2 Current influenza vaccination schedule in children undergoing treatment for cancer

Currently, all children in Australia aged 6 months to 18 years undergoing immunosuppressive therapy such as treatment for cancer are recommended to receive inactivated influenza vaccine as per The Australian Immunisation Handbook, 10th Edition (8). These recommendations have been developed by the Australian Technical Advisory Group on Immunisation (ATAGI) and approved by the National Health and Medical Research Council (NHMRC) (9, 10).

The recommendations are supported by a number of international studies, including one that was conducted locally in Perth in 2010 and 2011 by Kotecha *et al.,* which demonstrated that the inactivated influenza vaccine is safe, immunogenic and provides clinical protection in children receiving treatment for cancer (11-24). Kotecha *et al.* also demonstrated a significantly more effective immune response to two doses of the inactivated influenza vaccine in children <9 years of age compared to one dose. Therefore a recommendation was made that all immunocompromised children <9 years of age undergoing therapy for cancer should receive two doses of the inactivated influenza vaccine. Immunocompromised children aged ≥9 years continue to receive one dose of the inactivated influenza vaccine.

1.3 Standard evaluation of influenza vaccine response

The rise in influenza-specific hemagglutinin inhibition (HI) antibody titers in pre- and post-vaccination serum is used as a measure of the immune response to the vaccine. Seroprotection in an individual is defined as a post-vaccination HI titer of ≥40. Seroconversion is defined as either a fourfold increase in HI antibody titer if the

pre-vaccination titer was ≥10 or a rise in HI titer from <10 to ≥40 following vaccination.

The Committee for Proprietary Medicinal Products (CPMP) have established criteria to determine whether the influenza vaccine is considered to elicit an effective overall immunogenic response in a population (25). According to these criteria, the influenza vaccine is considered effective if it meets one of the following three criteria: seroprotection in >70% of patients; seroconversion in >40% of patients; or a geometric mean fold increase (GMFI) >2.5.

In the study by Kotecha *et al.*, the inactivated trivalent influenza vaccine containing the H3N2, H1N1 and B strains was considered to be immunologically effective by satisfying the criteria for a GMFI increase of >2.5 for all three vaccine strains. However, the vaccine did not meet efficacy criteria for seroprotection (H3N2 55%, H1N1 61% and B strain 41%) and seroconversion for the B strain (H3N2 43%, H1N1 43%, B strain 33%). In comparison, a recent multicentre open label study of inactivated monovalent H3N2 vaccine in healthy children aged 6 months to 17 years achieved seroprotection in 74% and 88% of children in the 3-8 year and 9-17 year old cohorts; while 72% and 85% seroconverted, respectively. In the same study, children aged 6 to 35 months showed a much lower response in general. Seroprotection and seroconversion were achieved in 19% and 38% of those that received one and two doses of the inactivated H3N2 vaccine, respectively. A group of children aged 6 to 35 months received one or two double doses of the vaccine (an equivalent of one or two standard doses for children ≥3 years old). These children achieved seroprotection and seroconversion in 30% and 47% of study participants, demonstrating an enhanced response (26). Overall, the data suggests room for improvement with regards to vaccine efficacy and identifying the ideal method with which to measure vaccine response in immunocompromised children with cancer.

A number of variables predictive of vaccine immune response have previously been analysed including patient age, sex, type of cancer, treatment intensity, total number of lymphocytes and lymphocyte subsets, and immunoglobulin levels. Kotecha *et al.* identified that children receiving treatment for solid tumours mount a significantly higher immune response to the vaccine compared with those treated for an underlying haematological malignancy. This is in keeping with previous findings of suppressed immunoglobulin producing B-cells in patients treated with more myelosuppressive regimens, such as children with leukaemia (27). Additional factors identified as predicting seroconversion include higher white cell count, lymphocyte count or IgG levels; increasing age; induction phase of therapy in acute lymphoblastic leukaemia; and vaccination post completion of therapy (14, 18-20, 23, 27). These correlates are reflective of underlying immune function and further stress the importance of optimising the timing and schedule of influenza vaccination and response detection method in children who are being treated for cancer.

1.4 Enhanced influenza vaccination schedule and extended vaccine response evaluation

The primary objective of our study is to determine whether an enhanced influenza vaccine schedule could further improve the immune response to the inactivated influenza vaccine in children receiving treatment for cancer.

We hypothesise that introduction of a two-dose inactivated influenza vaccine schedule will enhance the immune response in immunocompromised children and adolescents ≥9 years of age undergoing therapy for cancer, compared to the current one-dose schedule. This is in keeping with findings of a few studies performed in adolescent and adult patients with secondary immune deficiencies such as HIV or treatment for haematological malignancies, where repeated influenza vaccination resulted in optimised seroprotection (28-30).

We also aim to test whether a three-dose schedule of the inactivated influenza vaccine will provide a superior immune response compared to the currently recommended two-dose influenza vaccine schedule in immunocompromised children <9 years of age undergoing therapy for cancer. Only one study has previously attempted to assess the benefit of a three-dose schedule in children with cancer, however the outcomes of this study are limited as it was conducted over 35 years ago in a small patient cohort (31).

We will evaluate the safety of the additional dose of influenza vaccine by closely monitoring and reporting adverse events (AEs), and appointing an independent Data Safety Monitoring Board (DSMB) to ensure study participant safety. For safety reasons, children aged 6 to 35 months will not receive the additional influenza vaccine until an initial safety run in phase determines safety and tolerability of the third dose in children aged 3 to <9 years of age.

The rise in influenza-specific HI antibody titers is utilised as the primary measure of immune response to the influenza vaccine. However, this may not be the optimal assay for response assessment in immunocompromised populations and the benefit of an additional vaccine response detection method has been suggested in several small studies which have assessed influenza vaccine response post haematopoietic stem cell transplantation (32-35). Therefore, in addition to the well-established analysis of humoral immune response by HI antibody titer rise, we will measure cell-mediated immune response to the influenza vaccine, with the aim of validating an additional measure for assessment of immunogenicity in response to the inactivated influenza vaccine in immunocompromised children receiving treatment for cancer.

Our secondary objective will be to further define variables that are predictive of vaccine induced immune response, including assessment of patient age, sex, type of disease/treatment and parameters of baseline immune function including B-cells, CD4 and CD8 T-cells, memory cells and immunoglobulin levels. This will expand on previously conducted studies by Kotecha *et al.* and others with the aim of optimising the timing of vaccination.

1.5 Summary

We aim to establish an evidence-based and safe influenza vaccine immunisation schedule for immunocompromised children and adolescents undergoing treatment

for cancer in Western Australia, ultimately leading to a reduction in influenza infection associated morbidity and mortality in this vulnerable population and consequently reduce healthcare associated expenditure. We also aim to develop and validate a complementary assay for monitoring vaccine response in the immunocompromised patient. Ultimately, we envisage positive outcomes to have a global impact, with the potential for recommendations from this project to be implemented into national immunisation guidelines worldwide.

2.0 STUDY OBJECTIVES

Specific hypothesis

A two-dose inactivated influenza vaccine schedule will produce an enhanced immune response in immunocompromised children and adolescents ≥9 years of age undergoing therapy for cancer, compared to the current one-dose schedule.

A three-dose inactivated influenza vaccine schedule will produce an enhanced immune response in immunocompromised children <9 years of age undergoing therapy for cancer, compared to the current two-dose schedule.

2.1 Safety objectives

- To evaluate the safety of an enhanced influenza vaccine schedule in children undergoing treatment for cancer by closely monitoring and reporting adverse events following immunisation (AEFIs). An independent advisory committee will be appointed to ensure study participant safety.

2.2 Efficacy objectives

2.2.1 Primary efficacy objectives

- To determine whether an enhanced influenza vaccine schedule improves the immune response to inactivated influenza vaccine in children undergoing treatment for cancer.

    a. In children ≥9 years of age - to determine whether two doses of influenza vaccine (given at least four weeks apart) are superior to one dose.
    b. In children <9 years of age - to determine whether three doses of influenza vaccine (each given at least four weeks apart) are superior to two doses.

- To determine whether measurement of cellular immune response to the inactivated influenza vaccine provides a better surrogate end point for vaccine efficacy than influenza-specific hemagglutinin inhibition (HI) antibody titers in immunocompromised children with cancer.

2.2.2 Secondary efficacy objectives

- To define variables predictive of immune response to the inactivated influenza vaccine in a multivariate analysis of patient age, sex, type of diagnosis/treatment and parameters of base-line immune function including total lymphocyte count, B-cell count, CD4 and CD8 T-cell count, NK cell count, memory cells and immunoglobulin levels.

- To define the clinical incidence of laboratory proven influenza infection within the study population and determine whether infection correlates with cellular and humoral immune responses.

2.3 Exploratory objectives

- To explore the kinetics of influenza vaccine immune response by measuring humoral and cellular immune response at various time points after vaccination.

3.0 STUDY ENROLMENT

3.1 Participant enrolment

Participants may be enrolled in the study once all eligibility requirements for the study have been met. Participants will be eligible for entry into the study on provision of written informed consent by the child's parent(s) or guardian(s).

3.2 Participant/Parent Information sheet and Consent form

Legal guardians will be encouraged to include their child in the study discussions. Separate Information sheets will be provided for parent(s)/guardian(s), children and adolescents of 13-18 years of age, and children aged 8-12 years. These will contain information about the purpose of the study and any associated procedures, risks, inconveniences and discomforts; as well as processing the results of the research study. The information sheets will be written at a level of comprehension appropriate for the participant's age.

The parent/guardian will not be asked to sign the Consent form immediately. The voluntary nature of the study participation will be stressed and the involved parties will be assured that should they choose not to participate, their treatment and relationship with the treating institution will not be compromised.

The Information sheet and Consent form have been presented and reviewed by the Haematology and Oncology Parent Advisory Group (HOPAG) at Perth Children's Hospital (PCH). They have been received with a positive feedback. Minor editorial suggestions have been incorporated in the documents.

3.3 Participant recruitment

Participants will be recruited at PCH within the Haematology and Oncology Unit, either in the Outpatient (Clinic 1H) or Inpatient (Ward 1A) setting depending on the individual participant's location during the recruitment process.

3.4 Participant registration

Each participant will be assigned a study ID number at the time of enrolment. All information collected during the research study will be de-identified. Re-identification will only be allowed for the CPI and AI's, and regulatory authorities at the time of the study audit.

3.5 Timing

Participants must be enrolled before vaccination and sample collection begins. All eligibility criteria must be met and a consent form must be signed by the parent/guardian prior to entering the study.

3.6 Participant withdrawal criteria

Participants have the right to withdraw from the study at any time point, in which case their routine vaccination will not be affected.

Eligibility criteria and clinical condition will be checked at every study visit to ensure participants remain eligible and are clinically fit for vaccination.

3.7 Number of participants

It is estimated that 80 subjects will be recruited over the study period. This is based on the incidence and prevalence of patients receiving diagnosis and treatment for cancer at Perth Children's Hospital Haematology and Oncology Department.

3.8 Duration of study

The duration of the study is defined as a 24-month study period, starting on 2nd February 2020 and finishing on 31st January 2022.

4.0 PARTICIPANT ELIGIBILITY CRITERIA

All eligibility criteria listed below are interpreted literally and cannot be waived. All clinical and laboratory data required for determining eligibility of a participant enrolled on the study will be available in each participant's research record which will serve as the source document for verification at the time of audit.

4.1 Inclusion criteria

4.1.1 Male or female children of ≥6 months and ≤18 years of age undergoing therapy for cancer or within 6 months of completion of treatment.

4.1.2 Children immunosuppressed as a result of cancer therapy. This includes chemotherapy and radiotherapy but excludes biological agents such as Rituximab.

4.1.3 Patients receiving the influenza vaccine outside the hospital will be included provided that the dose and type of vaccine corresponds to the one recommended by the Health Department.

4.2 Exclusion criteria

4.2.1 Known contraindication to influenza vaccine as defined by the Australian Immunisation Handbook 10th Edition.

4.2.2 Patients who have received immunoglobulins within the last three months preceding the influenza vaccination.

4.2.3 Patients with a history of Guillain-Barre syndrome post influenza vaccine.

4.2.4 Patients post autologous or allogeneic haematopoietic stem cell transplantation.

4.2.5 Patients with non-malignant haematological disease.

5.0 STUDY DESIGN

5.1 Type of study

This will be a prospective non-randomised, open label study.

5.2 Study interventions

Each visit will comprise of two main components:

1. A blood sample will be taken to perform the following tests:
    a. A full blood count (FBC) including the differential will be requested prior each vaccination. Vaccination will only proceed if absolute neutrophil count (ANC) is ≥ 0.5 x 10^9/L, as per the Australian Immunisation Handbook 10th Edition recommendations.
    b. Lymphocyte subsets (prior Visit 1 only). This is to measure the number of B-cells, CD4 and CD8 positive T-cells, NK cells and memory T and B-cells.
    c. IgG, IgA and IgM levels (prior Visit 1 only).
    d. Cellular response to the influenza vaccine.

e. HI antibody titer measurement.

2. Influenza vaccine will be given (with the exception of the exit visit) to the participant after a baseline medical check. Participants found to be physically unfit will have their vaccination postponed. A minimum 30 minute observation period will be provided to monitor for development of an acute adverse event (AE) to the vaccine.

Participant age, sex, primary oncological diagnosis and phase of treatment will be documented at the start of the study. A detailed medical history will be taken including history of previous influenza illness, influenza immunisation and allergy to egg.

A substantial effort will be made to schedule study visits to coincide with participants' regular follow up outpatient visits and appointments for chemotherapy. Where possible, blood samples will be obtained from accessed central venous access devices (CVAD) to spare participants from venepunctures solely for study purposes.

Study visits will be spaced by a minimum of 4 weeks and maximum of 6 weeks as per the Australian Immunisation Handbook 10th Edition recommendations regarding timing of influenza vaccination and to allow for flexibility in arranging suitable appointments for study participants.

Any AEs post influenza vaccination will be documented at each visit. Each participant's parent/guardian will also be contacted by telephone one week after the influenza vaccination to document any delayed AEs related to the influenza vaccine.

Participants who develop an influenza-like illness (ILI) during the study period will be asked to contact the study team, except in the case of a fever defined as an isolated temperature of ≥38.5°C or two temperatures of ≥38.0°C in 12 hours, when they would present to the Emergency Department as per the standard Oncology Department guidelines.

ILI is defined by the following criteria, occurring greater than 72 hours after influenza vaccine administration:

- Elevated temperature (>37.5°C) or a clear history of fever (chills, rigors)

  AND

- Presence of at least one respiratory symptom such as:
  - Cough
  - Sore throat
  - Rhinorrhoea

Participants reporting symptoms of an ILI will be asked to present for a review. A nasopharyngeal test will be collected for virological testing at the time of the visit should their symptoms be objectively confirmed by the study team. Patients who present to the Emergency Department with a fever will be admitted to the Oncology

ward as per the standard pathway and a nasopharyngeal test will be collected for virological testing.

5.3 Study visit schedule

5.3.1 Visit 1

- Review of signed Consent form
- Review of eligibility criteria
- Documentation of previous medical history
- Documentation of previous influenza illness
- Documentation of previous influenza vaccination

- Baseline blood sample taken (10 mls)
  - FBC + differential
  - Lymphocyte subset
  - IgG, IgA, IgM
  - Cellular response to influenza vaccine
  - HI antibody titer

- 1st dose of influenza vaccine given
  - Pre-vaccination medical check
  - Post-vaccination 30 minute observation
  - Acute AE(s) documented and reported (see Section 8.0 for details)

- 7-day post-vaccine check phone call for documentation and reporting of AEFI(s) (see Section 8.0 for details)

- Optional blood sample taken 2 weeks after vaccination (5 mL)
  - Cellular response to influenza vaccine
  - HI antibody titer

5.3.2 Visit 2

- Review of ongoing eligibility
- Documentation of influenza-like illness
- Delayed AE(s) documented and reported (see Section 8.0 for details)

- Blood sample taken (7 mls)
  - FBC + differential
  - Cellular response to influenza vaccine
  - HI antibody titer

- 2nd dose of Influenza vaccine given
  - Pre-vaccination medical check
  - Post-vaccination 30 minute observation
  - Acute AE(s) documented and reported (see Section 8.0 for details)

- 7-day post-vaccine check phone call for documentation and reporting of AEFI(s) (see Section 8.0 for details)

- Optional blood sample taken 2 weeks after vaccination (5 mL)
    - Cellular response to influenza vaccine
    - HI antibody titer

5.3.3 **Visit 3**

This is the exit evaluation visit for participants ≥9 years of age.
For these participants the following will be performed:

- Documentation of influenza-like illness
- Delayed AE(s) documented and reported (see Section 8.0 for details)
- Blood sample taken (5 mls)
    - Cellular response to influenza vaccine
    - HI antibody titer

For participants <9 years* of age, the following will be performed:

- Review of ongoing eligibility
- Documentation of influenza-like illness
- Delayed AE(s) documented and reported (see Section 8.0 for details)

- Blood sample taken (7 mls)
    - FBC + differential
    - Cellular response to influenza vaccine
    - HI antibody titer

- 3rd dose of Influenza vaccine given
    - Pre-vaccination medical check
    - Post-vaccination 30 minute observation
    - Acute AE(s) documented and reported (see Section 8.0 for details)

- 7-day post-vaccine check phone call for documentation and reporting of AEFI(s) (see Section 8.0 for details)

- Optional blood sample taken 2 weeks after vaccination (5 mL)
    - Cellular response to influenza vaccine
    - HI antibody titer

*children aged 6 to 35 months will only receive the 3rd influenza vaccine dose if an initial safety run in phase determines that the third dose of the vaccine is safe and tolerable in children aged 3 - <9 years of age (see Section 8.3.4)

5.3.4 **Visit 4**

This is the exit evaluation visit for participants <9 years of age.
For these participants the following will be performed:

- Documentation of influenza-like illness
- Delayed AE(s) documented and reported (see Section 8.0 for details)
- Blood sample taken (5 mls)
  - Cellular response to influenza vaccine
  - HI antibody titer

Participants will have the option for additional blood samples to be taken in between the scheduled study visits to evaluate the kinetics of the immune response. In this instance 5 mls of blood will be taken to measure cellular response and HI antibody titers. This will be optional and only performed if the participant's CVAD is being accessed for treatment or routine investigational purposes at the time.

6.0 VACCINE

6.1 Influenza vaccine

The influenza vaccine used during the study period will be subject to seasonal recommendations set by the ATAGI, the Australian Influenza Vaccine Committee (AIVC), and the World Health Organization (WHO). All vaccines must be registered by the Therapeutic Goods Administration (TGA) to meet eligibility criteria set by the Australian Department of Health.

Currently, the following vaccines are recommended:

FluQuadri Junior

For children aged ≥6 months to <3 years
Administration route: intramuscular injection
Sponsor: Sanofi-Aventis Australia
Quadrivalent inactivated influenza vaccine
Each 0.25 mL pre-filled syringe contains:
    7.5 μg hemagglutinin for each of the 4 recommended influenza virus strains
    ≤50 μg formaldehyde
    ≤125 μg octoxinol 9
    ≤0.5 μg ovalbumin

FluQuadri

For children aged ≥3 years
Administration route: intramuscular injection
Sponsor: Sanofi-Aventis Australia
Quadrivalent inactivated influenza vaccine
Each 0.5 mL pre-filled syringe contains:
    15 μg hemagglutinin for each of the 4 recommended influenza virus strains
    ≤100 μg formaldehyde

≤250 µg octoxinol 9
≤1 µg ovalbumin

Annual influenza vaccination is recommended before the influenza season starts. Influenza community circulation usually peaks between June and September in most parts of Australia. The influenza vaccine usually becomes available in March or April of each year.

If more than one influenza vaccine is recommended, these should be administered 4 weeks apart. The first vaccine should be administered as soon as it becomes available to ensure that there is enough time to receive the second (and third dose) before influenza circulation peaks.

6.2 Contraindications and precautions

The only absolute contraindication to influenza vaccine is anaphylaxis after a previous dose of any influenza vaccine or any of the components of the influenza vaccine.

Children or adolescents with a known egg allergy, including history of anaphylaxis to egg, can be safely vaccinated with influenza vaccines (36). Those with a history of anaphylaxis must receive their influenza vaccine in a medical facility with staff experienced in recognising and treating anaphylaxis, such as Perth Children's Hospital. These children need a minimum 30 minute check post vaccination.

Some studies suggest that patients receiving immunological treatment for cancer, such as the checkpoint inhibitors (PD-1 inhibitor, Nivolumab; PD-L1 inhibitor, Pembrolizumab; CTLA-4 inhibitor, Ipilimumab) may have a higher risk of immune-mediated adverse events following the influenza vaccination (37). A more recent study on patients receiving a single checkpoint inhibitor has not confirmed these findings (38). Currently, the decision on proceeding with influenza vaccination in patients receiving immune checkpoint inhibitors is at the discretion of the treating Oncologist.

All influenza vaccines available in Australia are inactivated vaccines, so they cannot cause influenza. Nonetheless, post-vaccination symptoms can mimic influenza infection. 1-10% of people who receive a standard inactivated influenza vaccine develop fever, malaise and/or myalgia. These adverse events can start a few hours after vaccination and may last for 1-2 days.

7.0 LABORATORY EVALUATIONS

7.1 Assessment of humoral response to influenza vaccine

Blood samples obtained for the purpose of the humoral response to the influenza vaccine assessment will be centrifuged and sera stored at -80°C. At the end of the influenza season, predicted to be in October, the samples will be sent to the Victorian Infectious Diseases Reference Laboratory (VIDRL) at the Peter Doherty

Institute for Infection and Immunity in Melbourne, Victoria. This centre is a WHO Collaborating Centre for Reference and Research on Influenza and is part of the WHO Global Influenza Surveillance Network organisation (WHO GISN).

HI antibody titers against each vaccine strain will be measured in each sample by standardised assay developed and updated by the WHO GISN (39).

7.2 Assessment of cellular response to influenza vaccine

The cellular response to the influenza vaccine will be assessed in Dr Kotecha's laboratory at the Telethon Kids Institute (TKI). Levels of IFN-γ produced by T lymphocytes stimulated by influenza vaccine concentrate *in vitro* will be measured using sandwich immune-enzyme technology (ELISpot).

7.3 Laboratory testing for influenza infection

Nasopharyngeal swabs obtained from patients with ILI will undergo direct immunofluorescence testing for a range of respiratory viruses including influenza A and B. If negative, real-time PCR assay will be performed targeting matrix genes of Influenza A and B.

Nasopharyngeal swabs positive for Influenza A or B will be sent to VIDRL for influenza strain typing.

8.0 SAFETY, ADVERSE EVENT MONITORING AND REPORTING

8.1 Adverse events following immunisation (AEFIs)

8.1.1 Definition and Grading of AEFIs

Adverse events following immunisation are defined as unwanted or unexpected events following the administration of a vaccine. AEFIs also include conditions that may occur following the incorrect handling and/or administration of a vaccine.

The intensity of AEFIs will be graded on a three-point scale – mild, moderate, severe. For purposes of consistency, the intensity grades are defined as:

- Mild Does not interfere with subject's usual function.
- Moderate Interferes to some extent with subject's usual function.
- Severe Interferes significantly with subject's usual function.

8.1.2 Relationship of AEFIs to the influenza vaccine

To ensure consistency of AEFIs causality assessment, investigators will apply the following general guideline:

- Related event: There is a plausible temporal relationship between the onset of the AE and administration of the influenza vaccine, and the AE cannot be readily explained by the subject's clinical state, intercurrent illness, or concomitant therapies.

- Unrelated event: Evidence exists that the AE has aetiology other than the influenza vaccine and/or the AE has no plausible temporal relationship to the influenza vaccine administration.

- Expected event: An AE that is commonly reported by drug trial participants and listed in the product information brochure.

- Unexpected event: An unexpected event is an AE not listed in the product information brochure. This includes AEs for which the specificity of severity is not consistent with the description in the product information brochure.

8.1.3 Definition of a mild and moderate AEFI

A mild and moderate AEFI is an adverse event following the influenza vaccine that is expected and of a mild to moderate grade. The most commonly reported mild and moderate AEFIs in are listed below:

1. **Local:**

    - Tenderness
        - Mild - hurts if gently touched (subject whimpers, winces, protests or withdraws)
        - Moderate - hurts if gently touched with crying
    - Erythema
        - Mild - 0.5 to 2.0 cm
        - Moderate - >2.0 cm to 7.0 cm
    - Swelling/induration
        - Mild - ≤1.0 cm
        - Moderate - 1.0 to 5.0 cm

2. **Systemic:**

    - Fever
        - Mild - 38.0 to 39.0 °C
        - Moderate - >39.0 - 40.0 °C
    - Irritability
        - Mild - easily consolable
        - Moderate - requiring increased attention
    - Drowsiness
        - Mild - increased or prolonged sleeping bouts
        - Moderate - slightly subdued interfering with daily activity

- Decreased appetite
    - Mild - decreased interest in eating
    - Moderate - decreased oral intake
- Vomiting
    - Mild - easily tolerated by the participant, causing minimal discomfort and does not interfere with everyday activities
    - Moderate - sufficiently discomforting to interfere with normal everyday activities
- Diarrhoea
    - Mild - easily tolerated by the participant, causing minimal discomfort and does not interfere with everyday activities
    - Moderate - sufficiently discomforting to interfere with normal everyday activities
- Myalgia
    - Mild muscle pain
    - Moderate muscle pain, limiting instrumental ADL but not self-care
- Headache
    - Mild headache
    - Moderate headache, limiting instrumental ADL but not self-care

8.1.4 **Definition of a serious AEFI**

A serious AEFI is an AE at least possibly related to the study vaccine that is:

- life-threatening
- requires inpatient hospitalization or prolongation of existing hospitalization*
- results in persistent or significant disability/incapacity
- results in death

* Serious AEFI in the case of hospitalization or prolongation of existing hospitalization for fever is defined as hospitalization or prolongation of existing hospitalization with fever >40.5 °C

Examples of serious AEFIs are:

1. **Anaphylaxis** - anaphylaxis following immunization has been reported, but generally occurs very rarely. In the case of influenza vaccine, these probably represent an allergic response to a residual component of the manufacturing process, most likely egg protein. Anaphylaxis to egg is not a contraindication to administration of the influenza vaccine. The only absolute contraindication to influenza vaccine is anaphylaxis after a previous dose of any influenza vaccine or any of the components of the influenza vaccine.

2. **Hypotonic-hyporesponsive episode (HHE)** - HHE is a sudden onset of pallor and cyanosis, muscle hypotonia and reduced responsiveness or unresponsiveness occurring after vaccination, where no other cause is evident such as a vasovagal episode or anaphylaxis. The episode usually

occurs 1 - 48 hours after vaccination and resolves spontaneously. There are no known long-term side effects of HHE.

3. **Febrile convulsion** - febrile convulsions are rare following immunisation. They do occur more commonly, but still at a low rate, after some vaccines. For example, MMR and MMRV vaccines are associated with an increased risk of febrile convulsions approximately 7 to 12 days after the 1st vaccine dose (40, 41).
   In 2010, higher rates of fever and febrile convulsions occurred in children aged <5 years after influenza vaccination, especially in children aged <3 years. These were specifically associated with the vaccines Fluvax and Fluvax Junior at a rate of 4.4 per 1000 doses, compared with no such events reported among children who received an alternative vaccine in the same year (42). As a consequence, Fluvax and Fluvax Junior vaccines are no longer available in Australia.

8.2 AEFI(s) Evaluation Timeline

AEFI reporting will take place from the time the participant receives their first influenza vaccine until four weeks following the last vaccine administration.

Immediate AEFI(s) will be documented at the time of influenza vaccine administration. Delayed AEFI(s) will be checked one week after and up until the time point of the following vaccine administration four to six weeks later. Participants will be provided with a 7-day Side Effect Diary to facilitate documentation and recording of AEFIs (see Appendix II). Participants will be encouraged to report any AEFI(s) at any time during the study period.

8.3 Reporting and monitoring of AEFIs

8.3.1 **Reporting responsibilities**

Immunisation providers and medical practitioners in WA have a statutory requirement to notify AEFIs to the WA Department of Health through the WA Vaccine Safety Surveillance (WAVSS) System. The WAVSS reporting system is part of national reporting associated with the national network on Adverse Events Following Immunisation - Clinical Assessment Network (AEFI-CAN) (43).

Participants who receive their vaccine at the Stan Perron Immunisation Centre at PCH will be involved in automated surveillance with AusVaxSafety, a national vaccine safety initiative led by the National Centre of Immunisation Research and Surveillance (NCIRS) and funded by the Australian Department of Health.

Mild and moderate AEFIs do not require reporting to the WAVSS. These will be recorded, as well as any other AEFIs, by the study investigators as part of the Safety objectives evaluation.

A comprehensive list of AEFIs requiring reporting via the WAVSS System can be found in Appendix I. It includes the following entities:

- Abscess
- Acute flaccid paralysis
- Allergic reaction (generalised)
- Anaphylaxis
- Arthralgia
- Arthritis
- Brachial neuritis
- Death
- Encephalopathy
- Encephalitis
- Extensive limb swelling
- Fever >40.5 °C
- Guillain-Barré syndrome
- Hypotonic-hyporesponsive episode
- Injection site reaction (severe), including nodule
- Intussusception
- Lymphadenitis
- Meningitis
- Orchitis
- Osteitis
- Osteomyelitis
- Parotitis
- Severe and/or unusual rash
- Screaming
- Thrombocytopenia <50 x 10^9/L
- Toxic shock syndrome
- Vasovagal episode

8.3.2 **Required reporting criteria**

The following information regarding AEFI(s) will be recorded by the study investigators:

- Event description
- Event grade
- Event duration
- Relationship to the influenza vaccine (related or unrelated event)
- Event expectedness (expected or unexpected event)
- Action taken by the Study investigator
- Required medical attention

8.3.3 **Reporting Timelines**

All severe (non-mild, non-moderate) AEFIs will be reported within 48 hours. All serious AEFIs such as anaphylaxis, HHE and febrile convulsion will be reported within 24 hours of the event. Reports will be lodged via the WAVSS system as well as reported to the Data Safety Monitoring Board, to ensure participant safety.

8.3.4 **Data Safety Monitoring Board (DSMB)**

To ensure the safety of all study participants, a Data Safety Monitoring Board will be appointed. The DSMB will consist of five specialists from the following paediatric disciplines: Oncology, Haematology, Infectious diseases, Immunology and Neurology; as well as a Statistician.

The DSMB will receive a report issued by the study investigators every two weeks or at the completed recruitment of every 10 newly vaccinated study participants, regardless of the degree of possible AEs. In addition, the DSMB will receive a safety report after 6 participants have received the third dose of the vaccine in the 3 – <9 year age group and based on the safety outcomes of this report will provide a recommendation as to whether recruitment to the three dose schedule can commence in the 6-35 month age group.

Any unexpected, severe or serious AEFIs will be reported to the DSMB by the study investigators within the set timelines. The DSMB may recommend the study to be modified or terminated based on the reported events.

8.4 Stopping rules

A stopping rule will be met under the following circumstances:

- Where two subjects receiving the influenza vaccine develop a severe or serious AEFI, or an unexpected AEFI that is similar in nature and cannot be attributable to any other cause.

Once the stopping rule has been applied, enrolment of participants will be paused until the outcome of investigations by DSMB has been issued.

Study conduct activities such ongoing data entry, laboratory evaluations and participants follow up will continue during the pause.

Enrolment and vaccination may proceed at the discretion of a minimum of three DSMB members.

If the stopping rule has been triggered by a serious AEFI in two subjects within the same age group (i.e. anaphylaxis in two children <9 years old), additional influenza vaccine will not be administered to further study participants after this time point.

9.0 STATISTICS

9.1 Statistical design

This is a non-randomised open label study of influenza vaccine response to an enhanced immunisation schedule in a population of paediatric patients treated for cancer.

The primary aim will be to assess the within-patient differences in immune response after repeat doses of the inactivated influenza vaccine in children and adolescents who are being treated for cancer: for those ≥9 years of age comparing two doses to one dose, and for those <9 years of age comparing three doses to two doses.

Two separate methods of immune response evaluation will be assessed:

1. The humoral response will be assessed by measuring HI antibodies in participants' sera produced by stimulated B-lymphocytes in response to the influenza vaccine.
2. The cellular response will be assessed by measuring INF-γ produced by stimulated T-lymphocytes *in vitro* in response to an influenza vaccine concentrate.

One of the secondary aims will be to assess variables predictive of influenza vaccine immune response. The variables will include participant age, sex, type of disease/treatment and parameters of baseline immune function including total lymphocyte count, B-cell and T-cell count, NK cell count, memory cell count and immunoglobulin levels.

9.2 Sample size estimation

Sample size calculations have been based on differences in the paired proportions of vaccinated children demonstrating immunogenicity (seroprotection and seroconversion) after the standard number of doses and the extra dose, respectively. If the immunogenicity status of 15% of participants changes over the two time-points, 60 participants would have 80% power to detect an increase of 13.7% in the seroprotection and seroconversion rates and 80 participants would have 80% power to detect an increase of 12.0% (McNemar test, α=0.05). If discordancy in the immunogenicity status is observed in 20% of participants, the study would have 90% power to detect differences in rates of 18.1% and 15.8% for 60 and 80 participants, respectively. These rate increases would seem reasonable given increases achieved in healthy children after fewer doses.

9.3 Statistical analysis

The primary immunogenicity endpoints will be the percentage of participants achieving seroprotection and seroconversion. To assess if the extra vaccination dose improves immunogenicity, a McNemar test will be used to assess the

difference in these percentages as calculated from the immunogenicity status obtained after the last two doses of each individual. We will also evaluate geometric mean titers of HI antibodies at baseline and after vaccination. The geometric mean fold increase (GMFI), with 95% confidence intervals, will be calculated for each strain, using a log-normal approximation for the distribution of HI antibody levels pre and post vaccination.

Multivariate logistic regression models will be used to assess the influence of clinically relevant demographic and immunological variables to predict seroconversion to each influenza vaccine strain and complete seroconversion to all strains of the vaccine. Linear regression analyses of log-transformed HI antibody titers and log-transformed T-lymphocyte IFN-γ levels will be undertaken to assess the influence of clinically relevant variables on humoral and cellular responses to vaccination, respectively.

10.0 RECORD AND SAMPLE MANAGEMENT

10.1 Record keeping and handling

The REDCap application located at the Telethon Kids Institute (TKI) will be used for study participants' record keeping and handling. This is a secure web-based application used within academia for the capture, management and storage of health research data. Access to the REDCap is via a 3-tiered access of username and password protected login system. Only research investigators who have been granted project permission to the REDCap database by project custodians will have access, allowing for security and confidentiality of data stored.

Access to the back-end server is restricted to authorised system administrators within the TKI. Secure access to the TKI is maintained via logged key-card entry with an additional key card entry for the server room. All system administrators have current Good Clinical Practice (GCP) accreditation.

It is intended that the summary of analysed data will be submitted for a publication in a peer reviewed journal. The identity of study participants in publications and scientific presentations will be anonymised.

10.2 Sample collection and shipping

Each participant will be given a coded identifier, which will be attached to the participant source document. Only the coded identifier, the participant's initials and the collection date will appear on sample labels.

Sera obtained for the purpose of HI antibody measurement and nasopharyngeal swabs positive for Influenza A or B will be stored at -80°C. At the end of the influenza season the samples will be placed in biohazard bags and placed in boxes containing cool packs for shipment to the following address:

Peter Doherty Institute for Infection and Immunity
792 Elizabeth Street
Melbourne VIC 3000
Tel: 03 9342 9300
Fax: 03 9342 9329

11.0 QUALITY CONTROL AND QUALITY ASSURANCE

The project will be conducted in compliance with the protocol, Good Clinical Practice and regulatory requirements.

Influenza vaccines used during the study period will comply with the recommendations set by the ATAGI and approved by the NHMRC. All influenza vaccines used during the research study will be registered by the TGA.

12. ETHICAL AND REGULATORY CONSIDERATIONS

12.1 Ethics

The scientific content and clinical implications of the project will be considered by the Scientific Advisory Sub-Committee (SASC) prior to review by the Child and Adolescent Health Service (CAHS) Human Research Ethics Committee (HREC) and Research Governance Office (RGO).

12.2 Project monitoring

The study investigators will permit project related monitoring, audits and regulatory inspections with a direct access to the source documents.

The study progress and results will be presented in a form of a report/presentation to the WACPCN Fellowship committee, the sponsor of the CPI. The study progress will be presented to the HOPAG during and at completion of the project to ensure relevant research outcomes are disseminated to the community. Study participants will receive a letter summarising the results of the research study once all data have been analysed.

APPENDIX

APPENDIX I

Adverse events following immunisation requiring reporting via the WAVSS system

**Abscess**	Occurrence of a fluctuant or draining fluid-filled lesion at the site of injection, with or without fever.
**Acute flaccid paralysis***	Acute onset of flaccid paralysis of one or more limbs following any vaccine.
**Allergic reaction** (generalised)	A non-anaphylactic, generalised reaction characterised by 1 or more symptoms or signs of skin and/or gastrointestinal tract involvement WITHOUT respiratory or cardiovascular involvement.
**Anaphylaxis**	A rapidly evolving generalised multi-system allergic reaction characterised by 1 or more symptoms or signs of respiratory and/or cardiovascular involvement AND involvement of other systems such as the skin or gastrointestinal tract.
**Arthralgia**	Joint pain without redness or swelling.
**Arthritis**	Joint pain with redness and/or swelling.
**Brachial neuritis**	Pain in arm causing persisting weakness of limb on side of vaccination.
**Death**	Any death of a vaccine recipient temporally linked to vaccination, where no other clear cause of death can be established.
**Encephalopathy***	An acute onset of major neurological illness temporally linked with vaccination and characterised by any 2 or more of the following 3 conditions: seizures, severe alteration in level of consciousness or mental status (behaviour and/or personality) lasting for 1 day or more, and/or focal neurological signs which persist for 1 day or more.
**Encephalitis***	Characterised by the above-mentioned symptoms and signs of cerebral inflammation and, in many cases, CSF pleocytosis and/or virus isolation.
**Extensive limb swelling**	With or without redness which extends from the joint above to the joint below the injection site, or beyond a joint (above or below the injection site), or results in the circumference of the limb being twice the normal size.
**Fever**	Only very high fever should be reported, e.g. >40.5°C.
**Guillain-Barré Syndrome (GBS)***	Acute onset of rapidly progressive, ascending, symmetrical flaccid paralysis, without fever at onset of paralysis and with or without sensory loss. Diagnosed by cerebrospinal fluid investigation showing dissociation between cellular count and protein content.
**Hypotonic–hyporesponsive episode (shock, collapse)**	The sudden onset of pallor or cyanosis, limpness (muscle hypotonia), and reduced responsiveness or unresponsiveness occurring after vaccination, where no other cause is evident such as a vasovagal episode or anaphylaxis. The episode usually occurs 1 to 48 hours after vaccination and resolves spontaneously.
**Injection site reaction** (severe)	Reaction (redness and/or swelling) at site of injection which persists for more than 3 days AND is associated with ongoing symptoms such as pain or an inability to use the limb (see 'Brachial neuritis' above), and does not fulfil the case definition for extensive limb swelling and

	requires hospitalisation.
**Intussusception***	The invagination of a proximal segment of bowel into the distal bowel lumen.
**Lymphadenitis***<br>*(includes suppurative lymphadenitis)*	Occurrence of either at least 1 lymph node, 1.5 cm in diameter or larger, or a draining sinus over a lymph node.
**Meningitis***	Acute onset of major illness with fever and often neck stiffness/positive meningeal signs (Kernig, Brudzinski) and with CSF pleocytosis.
**Nodule**	A discrete or well demarcated soft tissue mass or lump that is firm and is at the injection site in the absence of abscess formation, warmth and erythema.
**Orchitis**	Swelling with pain and/or tenderness of testes.
**Osteitis**	Inflammation of the bone due to vaccination.
**Osteomyelitis**	Proven bacterial infection of bone.
**Parotitis**	Swelling and/or tenderness of parotid gland or glands.
**Rash**	Severe or unusual rash.
**Screaming**<br>(persistent)	The presence of crying which is continuous and unaltered for longer than 3 hours.
**Seizure**	Witnessed sudden loss of consciousness and generalised, tonic, clonic, tonic-clonic, or atonic motor manifestations.<br>• febrile seizures: with fever ≥38.5°C,<br>• afebrile seizures: without fever,<br>• syncopal seizures: syncope/vasovagal episode followed by seizure(s).
**Thrombocytopenia**	Platelet count <50 x 10^9/L.
**Toxic shock syndrome***	Abrupt onset of fever, vomiting, watery diarrhoea and shock within a few hours of vaccination which can be associated with other conditions listed here.
**Vasovagal episode**<br>(syncope, faint)	Episode of pallor and unresponsiveness or reduced responsiveness or feeling light headed AND occurring while vaccine being administered or shortly after (usually within 5 minutes), AND bradycardia, AND resolution of symptoms with a change in position (supine position or head between knees or limbs elevated).
**Other severe or unusual events**	Any unusual event that does not fit into any of the categories listed above, but is of medical or epidemiological interest, should be reported with a detailed description of the clinical features

* Diagnoses must be made by a physician

APPENDIX II

7-Day Side effect Diary

Name __________________

Please mark your child’s symptoms 1, 2, or 3

1 = Mild
2 = Moderate
3 = Severe

Symptoms	Day 0	Day 1	Day 2	Day 3	Day 4	Day 5	Day 6
Local reaction - pain							
Local reaction - redness							
Local reaction - swelling							
Fever*							
Irritability							
Drowsiness/Sleepiness							
Decreased appetite							
Vomiting							
Diarrhoea							
Muscle pain							
Headache							
Other							

*****In case of fever – defined as one single temperature of ≥38.5°C OR two temperatures between 38.0 and 38.5 within 12 hours, contact the Oncology HOT number and present to the ED at PCH

If any of the symptoms are marked as 3 or if you have any concerns, please call the study investigators on 6456 4420.

Side effects grading

Local symptoms

- Tenderness
  - Mild - hurts if gently touched (subject whimpers, winces, protests or withdraws)
  - Moderate - hurts if gently touched with crying
- Erythema
  - Mild - 0.5 to 2.0 cm
  - Moderate - >2.0 cm to 7.0 cm
- Swelling/induration
  - Mild - ≤1.0 cm
  - Moderate - 1.0 to 5.0 cm

Systemic symptoms

- Fever
  - Mild - 38.0 to 39.0 °C
  - Moderate - >39.0 - 40.0 °C
- Irritability
  - Mild - easily consolable
  - Moderate - requiring increased attention
- Drowsiness
  - Mild - increased or prolonged sleeping bouts
  - Moderate - slightly subdued interfering with daily activity
- Decreased appetite
  - Mild - decreased interest in eating
  - Moderate - decreased oral intake
- Vomiting
  - Mild - easily tolerated by the participant, causing minimal discomfort and does not interfere with everyday activities
  - Moderate – sufficiently discomforting to interfere with normal everyday activities
- Diarrhoea
  - Mild - easily tolerated by the participant, causing minimal discomfort and does not interfere with everyday activities
  - Moderate - sufficiently discomforting to interfere with normal everyday activities
- Myalgia
  - Mild muscle pain
  - Moderate muscle pain, not limiting self-care
- Headache
  - Mild headache
  - Moderate headache, not limiting self-care

REFERENCES

1. Smith MA, Altekruse SF, Adamson PC, Reaman GH, Seibel NL. Declining childhood and adolescent cancer mortality. Cancer. 2014;120(16):2497-506.
2. Baade PD, Youlden DR, Valery PC, Hassall T, Ward L, Green AC, et al. Population-based survival estimates for childhood cancer in Australia during the period 1997-2006. British journal of cancer. 2010;103(11):1663-70.
3. Cooksley CD, Avritscher EB, Bekele BN, Rolston KV, Geraci JM, Elting LS. Epidemiology and outcomes of serious influenza-related infections in the cancer population. Cancer. 2005;104(3):618-28.
4. Carr SB, Adderson EE, Hakim H, Xiong X, Yan X, Caniza M. Clinical and demographic characteristics of seasonal influenza in pediatric patients with cancer. The Pediatric infectious disease journal. 2012;31(11):e202-7.
5. Kempe A, Hall CB, MacDonald NE, Foye HR, Woodin KA, Cohen HJ, et al. Influenza in children with cancer. The Journal of pediatrics. 1989;115(1):33-9.
6. Elting LS, Whimbey E, Lo W, Couch R, Andreeff M, Bodey GP. Epidemiology of influenza A virus infection in patients with acute or chronic leukemia. Supportive care in cancer : official journal of the Multinational Association of Supportive Care in Cancer. 1995;3(3):198-202.
7. Tasian SK, Park JR, Martin ET, Englund JA. Influenza-associated morbidity in children with cancer. Pediatric blood & cancer. 2008;50(5):983-7.
8. The Australian Immunisation Handbook 10th Edition https://immunisationhandbook.health.gov.au/
9. Australian Technical Advisory Group on Immunisation https://www.health.gov.au/committees-and-groups/australian-technical-advisory-group-on-immunisation-atagi
10. National Health and Medical Research Council https://www.nhmrc.gov.au/research-policy/guideline-development
11. Kotecha RS, Wadia UD, Jacoby P, Ryan AL, Blyth CC, Keil AD, et al. Immunogenicity and clinical effectiveness of the trivalent inactivated influenza vaccine in immunocompromised children undergoing treatment for cancer. Cancer medicine. 2016;5(2):285-93.
12. Doganis D, Kafasi A, Dana H, Spanakis N, Baka M, Pourtsidis A, et al. Immune response to influenza vaccination in children with cancer. Hum Vaccin Immunother. 2018;14(9):2310-7.
13. Choi DK, Fuleihan RL, Walterhouse DO. Serologic response and clinical efficacy of influenza vaccination in children and young adults on chemotherapy for cancer. Pediatric blood & cancer. 2016;63(11):2011-8.
14. Ottoffy G, Horvath P, Muth L, Solyom A, Garami M, Kovacs G, et al. Immunogenicity of a 2009 pandemic influenza virus A H1N1 vaccine, administered simultaneously with the seasonal influenza vaccine, in children receiving chemotherapy. Pediatric blood & cancer. 2014;61(6):1013-6.
15. Carr S, Allison KJ, Van De Velde LA, Zhang K, English EY, Iverson A, et al. Safety and immunogenicity of live attenuated and inactivated influenza vaccines in children with cancer. The Journal of infectious diseases. 2011;204(10):1475-82.

16. Shahgholi E, Ehsani MA, Salamati P, Maysamie A, Sotoudeh K, Mokhtariazad T. Immunogenicity of trivalent influenza vaccine in children with acute lymphoblastic leukemia during maintenance therapy. Pediatric blood & cancer. 2010;54(5):716-20.
17. Reilly A, Kersun LS, McDonald K, Weinberg A, Jawad AF, Sullivan KE. The efficacy of influenza vaccination in a pediatric oncology population. Journal of pediatric hematology/oncology. 2010;32(5):e177-81.
18. Bektas O, Karadeniz C, Oguz A, Berberoglu S, Yilmaz N, Citak C. Assessment of the immune response to trivalent split influenza vaccine in children with solid tumors. Pediatric blood & cancer. 2007;49(7):914-7.
19. Matsuzaki A, Suminoe A, Koga Y, Kinukawa N, Kusuhara K, Hara T. Immune response after influenza vaccination in children with cancer. Pediatric blood & cancer. 2005;45(6):831-7.
20. Chisholm J, Howe K, Taj M, Zambon M. Influenza immunisation in children with solid tumours. European journal of cancer (Oxford, England : 1990). 2005;41(15):2280-7.
21. Porter CC, Edwards KM, Zhu Y, Frangoul H. Immune responses to influenza immunization in children receiving maintenance chemotherapy for acute lymphoblastic leukemia. Pediatric blood & cancer. 2004;42(1):36-40.
22. Hsieh YC, Lu MY, Kao CL, Chiang BL, Lin DT, Lin KS, et al. Response to influenza vaccine in children with leukemia undergoing chemotherapy. Journal of the Formosan Medical Association = Taiwan yi zhi. 2002;101(10):700-4.
23. Chisholm JC, Devine T, Charlett A, Pinkerton CR, Zambon M. Response to influenza immunisation during treatment for cancer. Archives of disease in childhood. 2001;84(6):496-500.
24. Feery BJ, Matthews RN, Evered MG, Gallichio HA. Antibody responses to influenza virus vaccine in patients with acute lymphocytic leukaemia. Australian paediatric journal. 1979;15(3):177-80.
25. Committee for Proprietary Medicinal Products (CPMP). Note for guidance on harmonisation of requirements for influenza vaccines. CPMP/BWP/214/96. European Agency for the Evaluation of Medicinal Products (EMEA). March 1997.
26. Munoz FM, Anderson EJ, Bernstein DI, Harrison CJ, Pahud B, Anderson E, et al. Safety and immunogenicity of unadjuvanted subvirion monovalent inactivated influenza H3N2 variant (H3N2v) vaccine in children and adolescents. Vaccine. 2019;37(36):5161-70.
27. Kersun LS, Reilly A, Coffin SE, Boyer J, Luning Prak ET, McDonald K, et al. A prospective study of chemotherapy immunologic effects and predictors of humoral influenza vaccine responses in a pediatric oncology cohort. Influenza and other respiratory viruses. 2013;7(6):1158-67.
28. de Lavallade H, Garland P, Sekine T, Hoschler K, Marin D, Stringaris K, et al. Repeated vaccination is required to optimize seroprotection against H1N1 in the immunocompromised host. Haematologica. 2011;96(2):307-14.
29. Haller W, Buttery J, Laurie K, Beyerle K, Hardikar W, Alex G. Immune response to pandemic H1N1 2009 influenza a vaccination in pediatric liver transplant recipients. Liver transplantation : official publication of the American Association for the Study of Liver Diseases and the International Liver Transplantation Society. 2011;17(8):914-20.
30. Hakim H, Allison KJ, Van de Velde LA, Tang L, Sun Y, Flynn PM, et al. Immunogenicity and safety of high-dose trivalent inactivated influenza vaccine compared to standard-dose vaccine in children and young adults with cancer or HIV infection. Vaccine. 2016;34(27):3141-8.

31. Brown AE, Steinherz PG, Miller DR, Armstrong D, Kellick MG, Gross PA, et al. Immunization against influenza in children with cancer: results of a three-dose trial. The Journal of infectious diseases. 1982;145(1):126.
32. Avetisyan G, Ragnavolgyi E, Toth GT, Hassan M, Ljungman P. Cell-mediated immune responses to influenza vaccination in healthy volunteers and allogeneic stem cell transplant recipients. Bone marrow transplantation. 2005;36(5):411-5.
33. Avetisyan G, Aschan J, Hassan M, Ljungman P. Evaluation of immune responses to seasonal influenza vaccination in healthy volunteers and in patients after stem cell transplantation. Transplantation. 2008;86(2):257-63.
34. Haining WN, Evans JW, Seth NP, Callaway GD, Wucherpfennig KW, Nadler LM, et al. Measuring T cell immunity to influenza vaccination in children after haemopoietic stem cell transplantation. British journal of haematology. 2004;127(3):322-5.
35. Guerin-El Khourouj V, Duchamp M, Krivine A, Pedron B, Ouachee-Chardin M, Yakouben K, et al. Cellular and humoral immunity elicited by influenza vaccines in pediatric hematopoietic-stem cell transplantation. Human immunology. 2012;73(9):884-90.
36. ASCIA Guidelines - Vaccination of the egg-allergic individual https://www.allergy.org.au/hp//papers/vaccination-of-the-egg-allergic-individual
37. Laubli H, Balmelli C, Kaufmann L, Stanczak M, Syedbasha M, Vogt D, et al. Influenza vaccination of cancer patients during PD-1 blockade induces serological protection but may raise the risk for immune-related adverse events. Journal for immunotherapy of cancer. 2018;6(1):40.
38. Wijn DH, Groeneveld GH, Vollaard AM, Muller M, Wallinga J, Gelderblom H, et al. Influenza vaccination in patients with lung cancer receiving anti-programmed death receptor 1 immunotherapy does not induce immune-related adverse events. European journal of cancer (Oxford, England : 1990). 2018;104:182-7.
39. WHO Global Influenza Surveillance Network Manual for the laboratory diagnosis and virological surveillance of Influenza https://www.who.int/influenza/gisrs_laboratory/manual_diagnosis_surveillance_influenza/en/
40. Klein NP, Fireman B, Yih WK, Lewis E, Kulldorff M, Ray P, et al. Measles-mumps-rubella-varicella combination vaccine and the risk of febrile seizures. Pediatrics. 2010;126(1):e1-8.
41. Jacobsen SJ, Ackerson BK, Sy LS, Tran TN, Jones TL, Yao JF, et al. Observational safety study of febrile convulsion following first dose MMRV vaccination in a managed care setting. Vaccine. 2009;27(34):4656-61.
42. Armstrong PK, Dowse GK, Effler PV, Carcione D, Blyth CC, Richmond PC, et al. Epidemiological study of severe febrile reactions in young children in Western Australia caused by a 2010 trivalent inactivated influenza vaccine. BMJ Open. 2011;1(1):e000016.
43. Adverse Events Following Immunisation - Clinical Assessment Network database for reporting of adverse events and clinical visits https://www.aefican.org.au/
